# A multidisciplinary approach to identify priority areas for the monitoring of a vulnerable family of fishes in Spanish Marine National Parks

**DOI:** 10.1186/s12862-020-01743-z

**Published:** 2021-01-21

**Authors:** Miquel Planas, Cristina Piñeiro-Corbeira, Carmen Bouza, Inés Castejón-Silvo, Manuel Vera, Marcos Regueira, Verónica Ochoa, Ignacio Bárbara, Jorge Terrados, Alexandro Chamorro, Rodolfo Barreiro, Jorge Hernández-Urcera, Irene Alejo, Miguel Nombela, Manuel Enrique García, Belén G. Pardo, Viviana Peña, Pilar Díaz-Tapia, Javier Cremades, Beatriz Morales-Nin

**Affiliations:** 1 Department of Ecology and Marine Resources, Instituto de Investigaciones Marinas (IIM-CSIC), Eduardo Cabello 6, 36208 Vigo, Spain; 2 BioCost Research Group, Facultad de Ciencias and CICA, Universidade da Coruña, 15071 Coruña, Spain; 3 Department of Zoology, Genetics and Physical Anthropology, Facultade de Veterinaria, Universidade de Santiago de Compostela, Campus de Lugo, Avenida Carballo Calero S/N, 27002 Lugo, Spain; 4 Instituto de Acuicultura, Universidade de Santiago de Compostela, Campus Vida s/n, 15782 Santiago de Compostela, Spain; 5 Mediterranean Institute for Advanced Studies (CSIC-UIB), 07190 Esporles, Spain; 6 Department of Marine Geosciences and Territorial Planning, Marine Sciences Faculty, University of Vigo, 36310 Vigo, Spain

**Keywords:** National park, Syngnathids, Habitat, Stable isotopes, Genetic identification, Conservation

## Abstract

**Background:**

Syngnathid fishes (Actinopterygii, Syngnathidae) are flagship species strongly associated with seaweed and seagrass habitats. Seahorses and pipefishes are highly vulnerable to anthropogenic and environmental disturbances, but most species are currently *Data Deficient* according to the IUCN (2019), requiring more biological and ecological research. This study provides the first insights into syngnathid populations in the two marine Spanish National Parks (PNIA—Atlantic- and PNAC—Mediterranean). Fishes were collected periodically, marked, morphologically identified, analysed for size, weight, sex and sexual maturity, and sampled for stable isotope and genetic identification. Due the scarcity of previous information, habitat characteristics were also assessed in PNIA.

**Results:**

Syngnathid diversity and abundance were low, with two species identified in PNIA (*Hippocampus guttulatus* and *Syngnathus acus*) and four in PNAC (*S. abaster*, *S. acus*, *S. typhle* and *Nerophis maculatus*). Syngnathids from both National Parks (NP) differed isotopically, with much lower δ^15^N in PNAC than in PNIA. The dominant species were *S. abaster* in PNAC and *S. acus* in PNIA. Syngnathids preferred less exposed sites in macroalgal assemblages in PNIA and *Cymodocea* meadows in PNAC. The occurrence of very large specimens, the absence of small-medium sizes and the isotopic comparison with a nearby population suggest that the population of *Syngnathus acus* (the dominant syngnathid in PNIA) mainly comprised breeders that migrate seasonally. Mitochondrial cytochrome b sequence variants were detected for *H. guttulatus*, *S. acus,* and *S. abaster*, and a novel 16S rDNA haplotype was obtained in *N. maculatus*. Our data suggest the presence of a cryptic divergent mitochondrial lineage of *Syngnathus* abaster species in PNAC.

**Conclusions:**

This is the first multidisciplinary approach to the study of syngnathids in Spanish marine NPs. Habitat preferences and population characteristics in both NPs differed. Further studies are needed to assess the occurrence of a species complex for *S. abaster*, discarding potential misidentifications of genus *Syngnathus* in PNAC, and evaluate migratory events in PNIA. We propose several preferential sites in both NPs for future monitoring of syngnathid populations and some recommendations for their conservation.

## Background

Syngnathidae is a singular fish family mostly inhabiting temperate and tropical sheltered, coastal marine waters [26, 47]. Seahorses and pipefishes utilize rocky, muddy, sandy, and rubble bottom habitats, generally associated with macrophytes communities [53]. Syngnathids are secondary consumers with specialized and opportunistic predatory strategies, ambushing small prey (mainly planktonic and nektonic crustaceans), showing a variety of diets, and foraging behaviours across genera and locations [53]. Seaweed and seagrass meadows promote the growth of most food sources and enhance the cryptic ability of syngnathids to avoid predators.

Syngnathids are valuable flagship species for conservation programs that will simultaneously benefit other fish [83]. Many species are vulnerable and threatened by habitat loss (pollution, sedimentation and eutrophication) and disturbances through boating and shipping [97], [43]. More than half of syngnathid species (two seahorse and eleven pipefish species) inhabiting Spanish coasts are currently classified as *Data Deficient*, and further research is needed to understand their biology and ecology (e.g., connectivity, migrations, mortality, etc.) [43].

Misidentifications of species have been reported due to cryptic morphology and unclear diagnostic traits among species, stressed by historical reference labelling errors in particular cases (e.g., European genus *Syngnathus*) [37, 102]. Genetic data are useful to solve taxonomic issues and complement morphological information, as a basic step towards the characterization and conservation of species and associated habitats [102]. Different mitochondrial markers have shown strong molecular support for species identification of seahorse and pipefish to clarify population and conservation studies (e.g., [51, 86, 100, 102].

Studies on syngnathids in the Iberian Peninsula are scarce and mainly focussed on specific topics for a reduced number of species [10, 16–19, 56, 94]. The present study is the first multidisciplinary approach for the global evaluation of syngnathid populations in Spanish coasts, particularly in marine National Parks (NP). Studies conducted in NPs would be highly valuable, considering their protection status and the supposed reduced impacts of most potential disturbances. Currently, there are two marine National Parks (NPs) in Spain, differing in their characteristics and biodiversity: Atlantic Islands National Park (PNIA) (Atlantic Ocean, NW Spain) and Cabrera Archipelago National Park (PNAC) (Balearic Islands, Mediterranean Sea). NPs should be the best marine ecosystems to ensure species survival and success in biodiversity conservation. However, protection requires a deep knowledge and analysis of habitats, values and threats, particularly for exceptional species and populations. In marine protected areas, there is the risk of a negative impact for syngnathids through increased predator abundance [39].

Marine ecosystems in PNIA host complex habitats and numerous ecological niches due to the extraordinary rich biota inhabiting soft and rocky floors typical of protected, semi exposed and exposed environments. Rocky shores are covered by seaweed, whereas the Western side is dominated by hard substrates covered by crusty, coralline and other turf-forming seaweed [69]. That side is exposed to Atlantic open water and extreme sea currents and waves, mainly in winter. The Eastern side is less exposed due to its position facing the Ría de Vigo. That side is characterized by a high biodiversity and productivity, and therefore it is an area of special interest for fishing. Such high productivity is promoted by important seasonal phytoplankton blooms [3, 77], and secondary production [9, 92], with high abundance in summer and seasonal changes in community structure. Copepods are largely predominant in winter, being accompanied in summer by other groups of fauna [9].

The fisheries system in PNIA is complex [13, 63, and the use of some types of fishing impacts negatively on syngnathids (by-catch and substrate degradation). Although areas of fishing are protected, they are not subject to special regulations [63]. Increasing tourism and nature activities promote public awareness for the conservation of marine ecosystems [69].

Cabrera Archipelago National Park (PNAC) is an IUCN category II Marine Protected Area (MPA) located 10 km southeast of Majorca (Balearic Islands, Mediterranean Sea), declared Spanish National Park in 1991. Algal beds, seagrass meadows and rocky bottoms dominate the subtidal zone. Three species of seagrass meadows are present: *Zostera noltei* (< 2 m depth), *Cymodocea nodosa* (0–25 m depth) and *Posidonia oceanica* (0–40 m depth).

Tourists visiting PNAC increases yearly, and recreational fishing and trawling in PNAC were banned in 1992. Small-scale fishing was regulated in 1995 but 80 small-scale boats from neighbouring towns continue fishing in some areas [57]. Fishing gears are regulated albeit overexploitation signs on the lobster trammel net fishery are evident [4].

The aims of this study were threefold. First, to assess distribution and habitat use of syngnathids in PNIA and PNAC (Additional file 1), each with highly distinctive environmental characteristics and vegetal assemblages. Second, to characterize syngnathid populations, which include the assessment of genetic identification and stable isotopes analyses. Finally, the unavailability of historical data for syngnathids in the Iberian Peninsula prevents the assessment of population trends. Hence, the third aim of this study was the selection of specific sites for further monitoring of distribution/abundance and temporal-seasonal patterns on important biological and ecological features (e.g., diet composition, animal migration). The results achieved would be valuable for the development of further conservation actions in both NPs.

## Results

### Habitat characterization in Cíes Archipelago (PNIA)

Soft bottom sediments were mostly coarse sandy (569 µm), with 90% sand and a prevalence of a single mode (Additional file 2). Muddy sands, with > 20% mud (< 63 µm), were only located in the deepest (17.6 m to 21 m) and distal areas of TR5, in the immediate vicinity of the muddy bottoms characteristics of the central part of Ría de Vigo. The presence of two or three mode samples in TR2, TR4 and TR3 reflected a mixture of particle sizes, including bioclastic gravel (bivalves and gastropods shells) and maerl elements. Different sedimentary environments (wide variability of textural characteristics) were present along some transects (e.g., TR3). Syngnathids were mostly sighted in sheltered sectors, preferring habitats with medium sands, better sorted and lacking mud (Additional file 2).

**Fig. 1:**
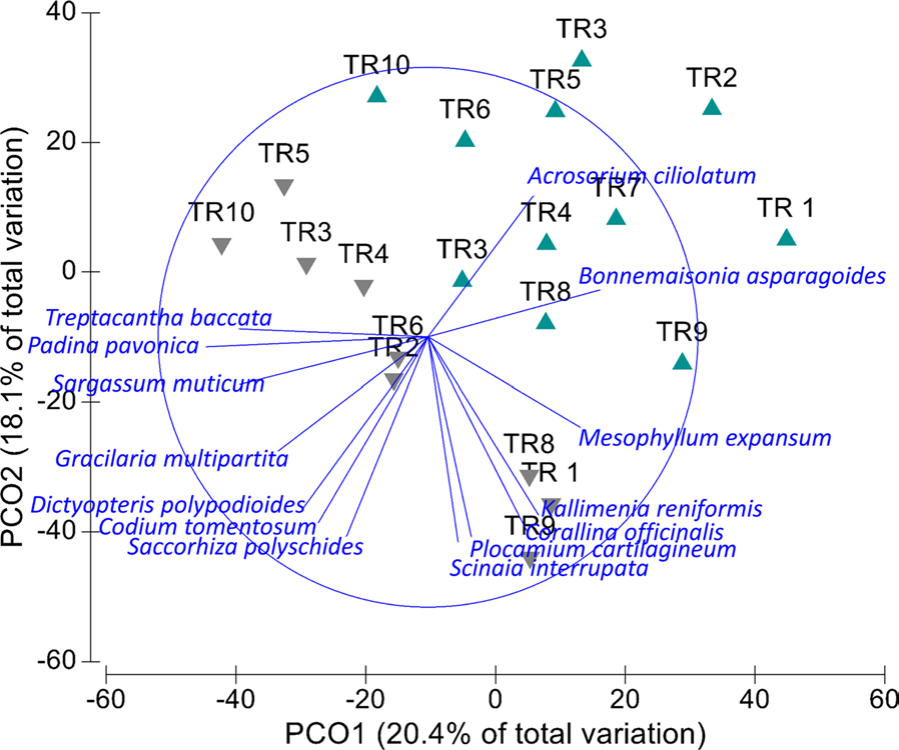
PNIA—Principal coordinates ordination of samples for *Transect* x *Season* pairwise combinations in seaweed assemblages on spring (green) and summer (grey). Overlay vectors are species whose cover has a Spearman correlation > 0.65 with any axis

Similarity of seaweed assemblages was analysed considering data of 55 species with medium–high abundance (Additional file 2, Additional file 3). Diversity (H’) and species richness (S) were particularly low in TR1, TR2, TR7 and TR10, especially in spring (Additional file 2). Seaweed cover increased in summer, especially in TR8 (633.8%) and TR9 (861.0%), but it was noticeable low in TR10 (42% in spring; 107% in summer) (Additional file 2). PERMANOVA results showed significant differences in assemblage structures for transects (df = 10; Pseudo-*F* = 1.3974; *P* = 0.0308) and seasons (df = 1; Pseudo-*F* = 3.711; *P* = 0.0031). Those differences are reflected in the two-dimensional PCOs plot (Fig. 1). Spring (left) and summer (right) samples followed a gradient along axis 1 (20.4% of total variation). Abundance increased in summer for most species, especially for *Treptacantha baccata, Padina pavonica, Corallina officinalis* or *Codium tomentosum* (strong negative correlation with PCO1; Spearman correlation > 0.65). Differences between transects were explained by axis 2 (18.1% of total variation), reflecting wave exposure. Transects TR1, TR8 and TR9 were clearly separated from the others, especially from TR10 and TR3. These results explained spatial differences between transects, with TR9, TR8 and TR7 as the most northern sites of Cíes Archipelago, and TR1 located in the west side of the southern island. The remaining transects (especially TR10) were located in areas less exposed to wave impact and current actions. Vectors overlay in PCO plot indicated that species such as *T. baccata, P. pavonica* or *C. tomentosum* were more abundant on less exposed areas, while *Mesophyllum expansum, C. officinalis*, *Plocamium cartilagineum* and *Kallymenia reniformis* preferred more wave-exposed sites (Spearman correlation > 0.65).

### Syngnathids in PNIA and PNAC

In PNIA, two species of syngnathids were identified morphologically and genetically: the long-snouted seahorse *Hippocampus guttulatus* Cuvier, 1829, and the greater pipefish *Syngnathus acus*, Linnaeus 1758. A total of 28 specimens were sighted in PNIA from 4 to 15 m depth (mostly at < 8 m), with six transects providing at least one fish (Table 1). None of the individuals marked in spring were recaptured in summer. All PCO showed a positive correlation of syngnathids with seaweed assemblages on transects TR3, TR4, TR5 and especially TR10 (Spearman correlation > 0.65) in summer (Additional file 2). The highest abundance (0.06–0.13 syngnathids 100 m^−2^) were recorded in mixed (sand-rock) or rocky substrates on transects TR3 and TR10 (32 and 43% of total specimens, respectively). Syngnathids were missing in the more exposed transects TR1, TR7, TR8 and TR9 (northern and southern areas with rocky substrate and coarse sand patches). TR1 was facing SW waves (prevalent component during storm winter conditions), while TR7, TR8 and TR9 were facing N waves (prevalent component during storm summer conditions). The most common species was *S. acus* (n = 24), which comprised 86% of total fish sighted.

**Table 1:** PNIA—Syngnathids (*Syngnathus acus* and *Hippocampus guttulatus*) captured in spring and summer 2016 surveys

Species	TR	Date	Depth (m)	SL (cm)	W (g)	Sex	Sexual state	Substrate
Spring 2016								
*S. acus*	2	4-may	15	23.8	8.4	Male	Pregnant	Gravel
	5	5-may	6	14.8	1.3	Female		Sandy
	6	5-may	5.5	44.0	64.7	Female	Ovigerous	Sandy
	6	5-may	6	34.2	27.2	Female	Ovigerous	Sandy
	10	20-may	4	32.0	23.2	Male	Pregnant	Rocky
	3	7-jun	5.5	44.9	66.7	Male	Pregnant	Sandy-Rocky
	3	7-jun	5.5	28.9	14.6	Female	Ovigerous	Sandy-Rocky
	3	7-jun	6	35.0	25.1	Female	Ovigerous	Sandy-Rocky
	3	7-jun	5	31.3	28.6	Male	Pregnant	Sandy-Rocky
	3	7-jun	5.5	24.5	6.2	Male	Pregnant	Sandy-Rocky
	3	7-jun	5.5	25.2	10.6	Female	Ovigerous	Sandy-Rocky
	3	7-jun	6	34.3	25.9	Female	Ovigerous	Sandy-Rocky
	3	7-jun	6	15.0	–	Female		Sandy-Rocky
	10	8-jun	4	49.7	62.5	Female	Ovigerous	Rocky
	10	8-jun	4	25.5	16.6	Male	Pregnant	Rocky
	10	9-jun	7	20.5	4.1	Female		Sandy
	10	9-jun	7.5	33.0	21.4	Male	Pregnant	Sandy
	10	9-jun	5	45.6	67.6	Male	Pregnant	Sandy-Rocky
*H. guttulatus*	10	9-jun	8.5	22.7	25.8	Female		Rocky
	10	9-jun	8.5	21.8	25.6	Male		Rocky
Summer 2016								
*S. acus*	2*	1-sep	–	–	–	–		
	3	6-sep	6	40.0	50.7	Female		Sandy
	4	6-sep	6.5	30.8	21.6	Male		Rocky
	4	6-sep	4	17.6	3.1	Female		Sandy
	10	7-sep	7.5	39.0	40.4	Female		Sandy-Rocky
	10	7-sep	7.5	42.9	58.6	Male		Sandy-Rocky
*H. guttulatus*	10	7-sep	8	19.5	21.3	Male		Rocky
	10	7-sep	8	18.3	14.8	Female		Rocky

*TR* transect, *SL* standard length, *W* wet weight

*Not captured

Most collected fishes were large adults, with *S. acus* averaging 31.8 ± 10.0 cm SL (range: 14.8–49.7 cm) and *H. guttulatus*, 22.6 ± 2.0 cm (range: 18.7–22.7 cm). Mean weights were 21.9 ± 5.2 g (range: 1.3–67.6 g) in *S. acus* and 22.6 ± 2.0 (range: 14.8–25.8 g) in *H. guttulatus*.

In *S. acus*, meristic features were: 20 trunk rings (range: 19–20), 42 tail rings (41–44), 12 pectoral fin rays (9–12), 38 dorsal fin rays (37–41), 3 anal fin rays and 10 caudal fin rays. Only four seahorses were observed (TR10; 8.0–8.5 m depth). The species showed positive allometry (b = 3.32) (Additional file 2), and lengths and weights in spring and summer did not differ significantly (Tukey HSD, n = 23, P = 0.519 for length, P = 0.471 for weight). Pregnant males and ovigerous females did not differ neither in length (Tukey HSD, n = 20, P = 0.464) nor weight (Tukey HSD, n = 20, P = 0.983). Abundance declined in summer (25%), when mature individuals were not observed. Contrarily, 90% of males and 70% of females collected in spring were pregnant (pouch carrying fertilized eggs/embryos) or ovigerous (full gonads with hydrated eggs), respectively. The minimum length recorded was 23.8 cm (8.4 g) in pregnant males and 25.2 cm (10.7 g) in ovigerous females.

**Fig. 2:**
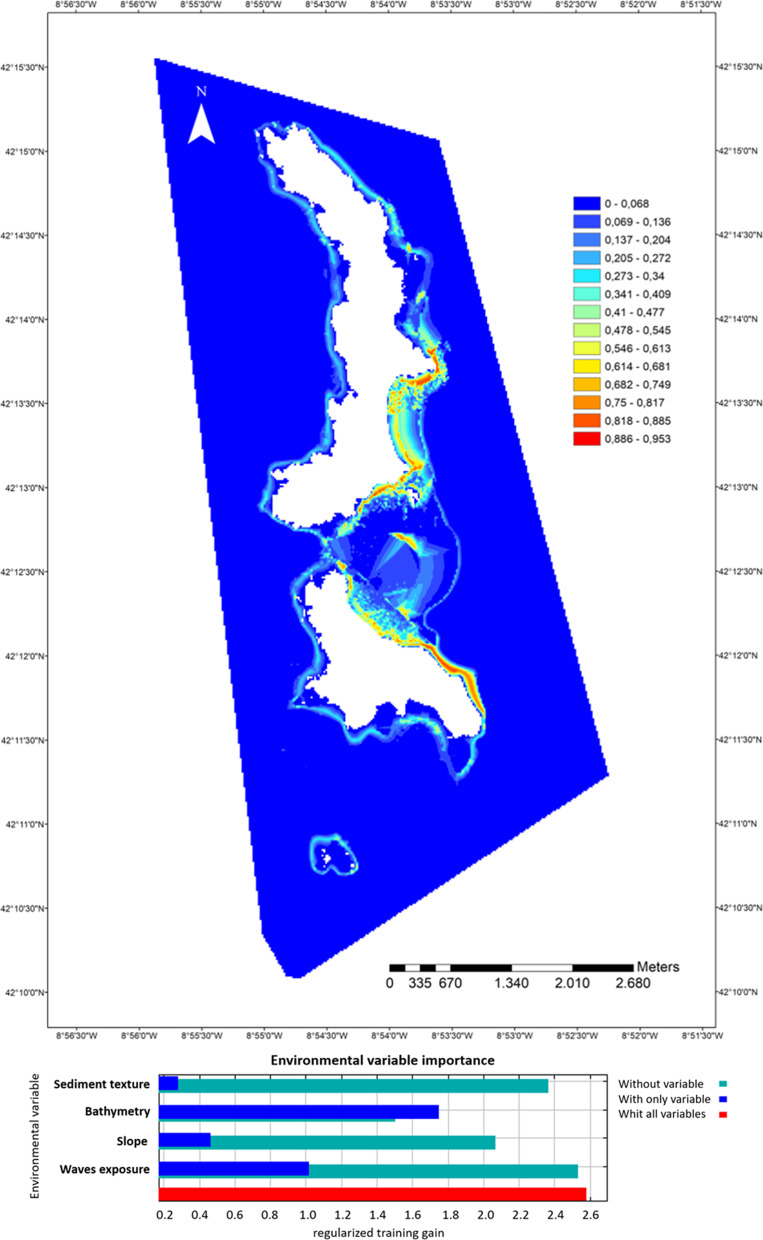
PNIA—Maxent habitat suitability map for syngnathids (pooled specimens of *S. acus* and *H. guttulatus*) in Cíes Archipelago. Environmental suitability is depicted using a color gradient from blue (low environmental suitability) to red (high suitability). The bottom panel shows the results of the jackknife test of variable importance training data

With regard to distribution of total syngnathids (*S. acus* and *H. guttulatus*) in Cíes Archipelago (PNIA), Maxent model achieved an AUC value of 0.98, indicating a very good degree of discrimination between the locations where the species were present and those where they were absent. Figure 2 shows the probability of habitat suitability for syngnathids. The model highlights higher probabilities of occurrence in a few limited areas (red color) on the East coast of the islands. Three of those areas (TR 3, TR4-5 and TR10) were selected for further monitoring of syngnathids in PNIA (see Discussion). The Jackknife test (i.e. variable importance) showed that bathymetry and wave exposure were the most influential variables (Fig. 2).

In PNAC, four pipefish species were morphologically identified but seahorses were lacking. Only three specimens (one *Syngnathus acus*, two *Nerophis maculatus* Rafinesque, 1810) were sighted on 37 visual censuses and 15 specimens (10 *Syngnathus abaster* Risso, 1827, two *Syngnathus typhle* Linnaeus, 1758, two *Syngnathus acus* and, one *Nerophis maculatus*) were captured in seven fishing sets (Table 2). All specimens were captured at 11–21 m depth, except for two *N. maculatus* (< 8 m depth). Occurrences in *C. nodosa* meadows (Es Burri) by fishing sampling and visual census were similar (1.3 and 1.2 syngnathids 100 m^−2^, respectively), but two-folds higher than by visual censuses in *P. oceanica* meadows and macroalgal beds in rocky substrates (0.03 individuals per 100 m^−2^).

**Table 2:** PNAC: Syngnathids captured in 2016 surveys and sampling information

Species	Site	Date	Depth (m)	SL (cm)	Benthic community	Sampling method
*Syngnathus acus*	Es Port	21 April	13.5–15	27.0	*Cymodocea nodosa*	VC, MC
	Es Burri	6 Sept	11–13	11.5	*Cymodocea nodosa*	TN
	Es Burri	8 Sept	13–15	4.4	*Cymodocea nodosa*	TN
*Syngnathus abaster*	Es Burri	6 Sept	11–13	7.6	*Cymodocea nodosa*	TN
	Es Burri	6 Sept	11–13	7.0	*Cymodocea nodosa*	TN
	Es Burri	8 Sept	13–15	9.0	*Cymodocea nodosa*	TN
	Es Burri	8 Sept	13–15	4.1	*Cymodocea nodosa*	TN
	Es Burri	1 Dec	11–13	10.3	*Cymodocea nodosa*	TN
	Es Burri	1 Dec	11–13	8.1	*Cymodocea nodosa*	TN
	Es Burri	1 Dec	11–13	9.9	*Cymodocea nodosa*	TN
	Es Burri	2 Dec	13–15	8.8	*Cymodocea nodosa*	TN
	Es Burri	2 Dec	13–15	9.8	*Cymodocea nodosa*	TN
	Es Burri	2 Dec	13–15	7.6	*Cymodocea nodosa*	TN
*Syngnatus typhle*	Es Burri	8 Sept	13–15	6.4	*Cymodocea nodosa*	TN
	Es Burri	8 Sept	13–15	6.4	*Cymodocea nodosa*	TN
*Nerophis maculatus*	Es Burri	23 April	6–8	nm	*Posidonia oceanica*	VC, MC
	Es Burri	8 Sept	13–15	10.0	*Cymodocea nodosa*	TN
	Es Burri	9 Sept	19–20	12.5	*Cymodocea nodosa*	VC, MC
	Santa María	1 Dec	4.4–6	nm	*Posidonia oceanica*	MC (outside VC)

*VC* visual census, *MC* manual capture, *TN* Trawl net (*gánguil*), *SL* standard length, *nm* not measured

### Genetic identification in syngnathids

Genetic samples from 33 syngnathid specimens morphologically identified in PNIA (22 *S. acus* and 4 *H. guttulatus*) and PNAC (6 *S. abaster* and 1 N*. maculatus*) were assayed. The marker cytochrome b (Cytb) was used to support the molecular identification of seahorse and pipefish species [100, 101]. The ribosomal mitochondrial marker 16S rDNA, which also supported molecular phylogeny in syngnathids [100], was assayed in the single sample of *N. maculatus* in which Cytb could not be amplified.

Length for Cytb sequences was 1149 base pairs (bp) in *S. acus*. In PNIA, nine Cytb haplotypes (12 variable sites) were detected (Additional file 2) and identified as *S. acus* (identity > 99.5% and e-value = 0.0), one of them (Cytb_SA13; GenBank Accession Number: MW080699) identical to the reference used for this species (AF356040; [100]. Haplotypes Cytb_SA01 (MW080694) and Cytb_SA02 (MW080695) were the most abundant (nine and six individuals, respectively), whereas the rest were only found in one individual (Cytb_SA07: MW080696,Cytb_SA10: MW080697; Cytb_SA11: MW080698; Cytb_SA14: MW080700; Cytb_SA16: MW080701; Cytb_SA17: MW080702), resulting in a haplotype diversity (h) of 0.7792 in the PNIA population sampled.

The four seahorse specimens studied were identified as *H. guttulatus*. Three Cytb haplotypes (564 bp) were detected (Cytb_HG01-03), comprising two variable sites (five when the reference sequence was included) (Additional file 2). Cytb_HG01, Cytb_HG02 and Cytb_HG03 were identical to *H. guttulatus* sequences reported across European populations [101]: KM061961 (GB10), KM061963 (GB7) and KM061980 (GB23), respectively. The most abundant *H. guttulatus* haplotype was Cytb_HG03 (two individuals), providing an h estimate of 0.8333.

**Fig. 3:**
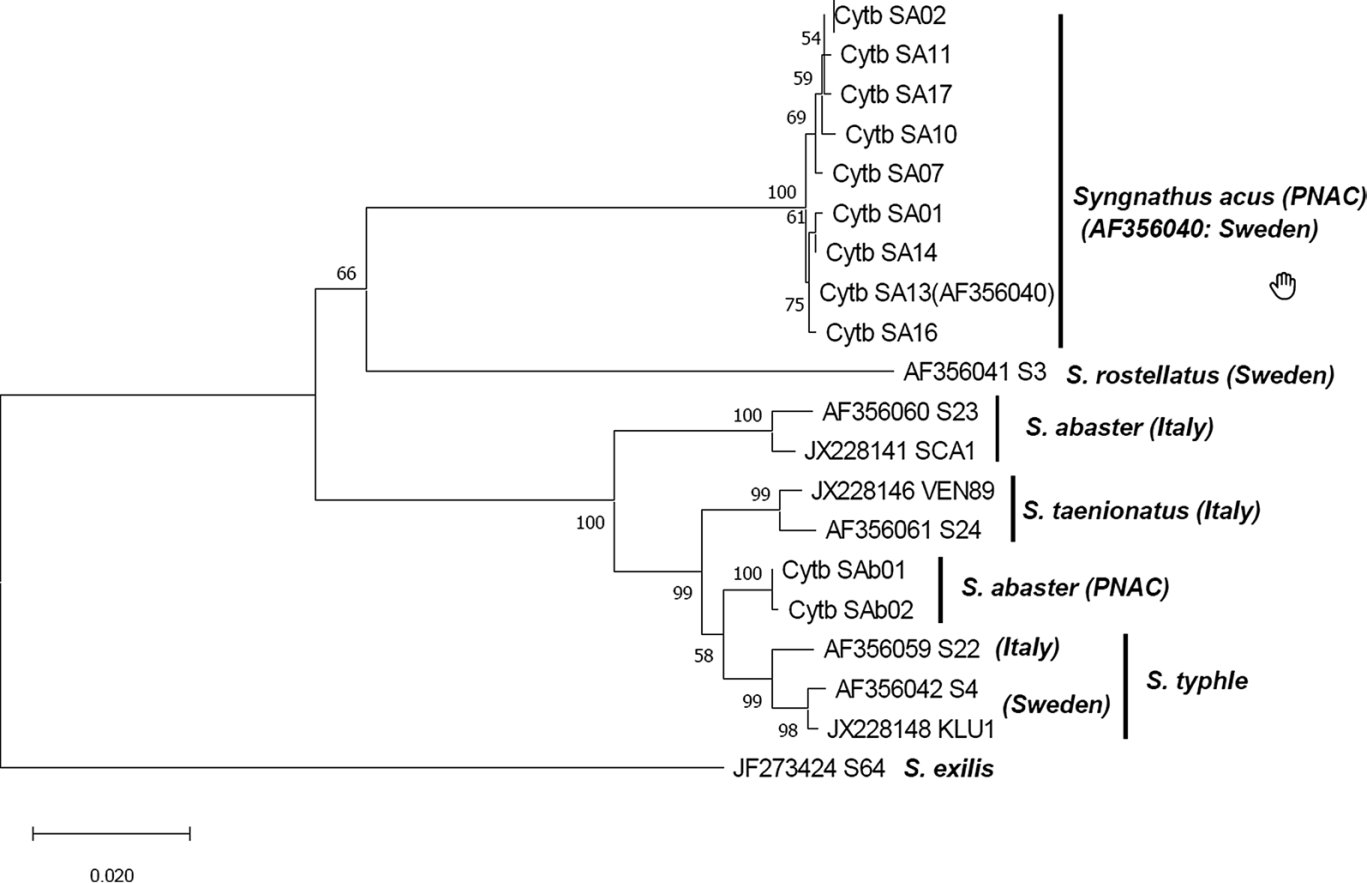
NJ tree (p-distance) for the *Syngnathus* genus. “SA” and “SAb” show *S. acus* and *S. abaster* haplotypes, respectively. Numbers on branches indicate the bootstrap value for their confidence (1000 replicates). GenBank reference sequences for *S. abaster* (AF356060_S23; JX228141_SCA1), *S. typhle* (AF356042_S4; AF356059_S22; JX228148_KLU1), *S. acus* (AF356040), *S. rostellatus* (AF356041_S3), *S. taenionatus* (AF356061_S24; JX228146_VEN89) and *S. exilis* (JF273424_S64) are also included. Following Mwale et al. [58], *S. exilis* was used as outgroup

For six specimens morphologically identified as *S. abaster*, two Cytb haplotypes were detected (Cytb_SAb01: MW080703 and Cytb_SAb02: MW080704) in four and two fish, respectively (h = 0.5333); showing 139 variable sites respect to the *S. acus* sequence (AF356040; Additional file 2). These two Cytb_SAb haplotypes showed a higher sequence identity with a *S. typhle* reference haplotype (JX228148; identities > 98%) than with other Cytb sequences of *S. abaster* (JX228141; identities ≤ 95%) available at GenBank database. Thus, net genetic distances between the groups formed by the Cytb_SAb haplotypes and the *S. typhle* haplotypes available at the GenBank (0.0140 ± 0.0033) was lower than the distance between the groups formed by the PNAC Cytb_SAb haplotypes and the *S. abaster* haplotypes from GenBank (0.0478 ± 0.0061). Phylogenetic analysis also corroborated these results. Thus the two Cytb_SAb haplotypes from PNAC were grouped in a monophyletic cluster clearly differentiated from GenBank Cytb sequences of *S. abaster* [58] and other pipefish species distributed in Mediterranean areas, more closely related to *S. typhle* and *S. taenionatus* than to *S. acus* and *S. rostellatus* (Fig. 3).

Failed amplification of Cytb was observed in the single genetic sample studied of *N. maculatus*, but a novel 16S rDNA haplotype of 521 pb (16S_NM01: MW080705) was detected for this pipefish, with 48 variable sites respect to a related reference species (*N. ophidion*; AF354994), in absence of available GenBank data for *N. maculatus*.

### Stable isotope signatures in syngnathids

In PNIA, *H. guttulatus* and *S. acus* (Table 3) differed significantly for δ^13^C (ANOVA, F_1,21_ = 0.492, P = 0.026) but not for δ^15^N (F_1,21_ = 5.744; P = 0.491). Isotopic values in *S. acus* ranged from -16.6 to -14.7 ‰ for δ^13^C (-16.1 ± 0.4) and from 9.1 to 11.9 ‰ for δ^15^N (10.8 ± 0.7), being correlated with fish size (Additional file 2). Season-sex comparisons showed inter-seasonal differences only for δ^13^C, with spring values (− 16.2 ± 0.3 ‰) lower than in summer (− 15.6 ± 0.5 ‰) (ANOVA, F_1,15_ = 9.52, P = 0.008), and lower values in males (− 16.2 ± 0.2 ‰) than in females (− 15.9 ± 0.6 ‰) (ANOVA, F_1,15_ = 6.65, P = 0.021). Sex-maturity comparisons showed similar δ^13^C values for mature (− 16.2 ± 0.3 ‰) and immature (15.9 ± 0.6 ‰) fishes (ANOVA, F_1,13_ = 2.88, P = 0.104) but lower δ^15^N signals in the later (11.1 ± 0.5 ‰ for mature, 10.4 ± 0.8 ‰ for immature) (ANOVA, F_1,13_ = 2.79, P = 0.014).

**Table 3:** PNIA—Mean (± sd) δ^13^C and δ^15^N values in *H. guttulatus* and *S. acus* sampled in spring and summer 2016 in Cíes Archipelago

		δ^13^C				δ^15^N			
Species	Season	Mean ± sd	Max	Min	n	Mean ± sd	Max	Min	n
*H. guttulatus*	Pooled	− 16.6 ± 0.2	− 16.3	− 16.8	4	11.0 ± 0.4	11.6	10.6	4
	Spring	− 16.5 ± 0.2	− 16.3	− 16.6	2	11.2 ± 0.5	11.6	10.9	2
	Summer	− 16.7 ± 0.2	− 16.5	− 16.8	2	10.8 ± 0.4	11.1	10.6	2
	♂ Spring	− 16.3 ± 0.0	− 16.3	− 16.3	1	11.6 ± 0.0	11.6	11.6	1
	♂ Summer	− 16.8 ± 0.0	− 16.8	− 16.8	1	11.1 ± 0.0	11.1	11.1	1
	♀ Spring	− 16.6 ± 0.0	− 16.6	− 16.6	1	10.9 ± 0.0	10.9	10.9	1
	♀ Summer	− 16.5 ± 0.0	− 16.5	− 16.5	1	10.6 ± 0.0	10.6	10.6	1
*S. acus*	Pooled	− 16.1 ± 0.4	− 14.7	− 16.6	21	10.8 ± 0.7	11.9	9.1	21
	Spring	− 16.2 ± 0.3	− 15.8	− 16.6	16	11.0 ± 0.7	11.9	9.8	16
	Summer	− 15.6 ± 0.5	− 14.7	− 16.1	5	10.3 ± 0.8	11.2	9.1	5
	♂ Spring	− 16.2 ± 0.3	− 15.9	− 16.6	8	11.1 ± 0.6	11.9	10.0	8
	♂ Summer	− 16.0 ± 0.1	− 16.0	− 16.1	2	10.1 ± 1.5	11.2	9.1	2
	♀ Spring	− 16.2 ± 0.3	− 15.8	− 16.6	8	10.9 ± 0.5	11.7	9.8	8
	♀ Summer	− 15.3 ± 0.5	− 14.7	− 15.7	3	10.3 ± 0.2	10.5	10.2	3

SIA in PNAC was only performed on a reduced number of *S. abaster* (n = 5; 7.6–10.3 cm length). Isotopic values were not correlated with fish size (Spearman correlation = − 0.3 and 0.1 for N^15^ and C^13^ respectively), ranging from − 15.2 to − 19.6 ‰ for δ^13^C (− 16.4 ± 1.8) and from 6.5 to 7.3 ‰ for δ^15^N (6.9 ± 0.3).

## Discussion

### Diversity, distribution and habitat of syngnathids

PNIA and PNAC differed in habitat characteristics and syngnathids occurrence. Sixteen syngnathid species are known in Europe [20] but only five were identified in our study. Two species were sighted in PNIA: the seahorse *H. guttulatus* (very low abundance) and the pipefish *S. acus* (Highly dominant). Most specimens sampled from PNIA were very large, lacking young or small sized fishes. In PNAC, seahorses were absent and four pipefish species (*S. abaster, S. acus, S. typhle, N. maculatus*) were recorded, comprising mostly small specimens. Syngnathids were considered uncommon in PNAC, though occurrences of *S. acus*, *S. typhle*, *H. guttulatus* and *H. hippocampus* were known [76]. Our results indicate low pipefish occurrences, with higher abundance in Es Burri Bay, particularly for *S. abaster*. This species is also the most common in other nearby areas (Mar Menor, SW Spain) [21]. *H. guttulatus* and *N. maculatus* are classified as *Data Deficient*, whereas the others are considered *Least Concern* [42]. In Balearic Islands, *S. abaster* is *Vulnerable*, *S. typhle* is *Near Threatened*, and *S. acus* and *N. maculatus* are *Least Concern* [31].

High congruence between genetic and morphological data for species identification was observed, except for *S. abaster* in PNAC respect to previous mitochondrial sequences for this species. In PNIA, eight novel Cytb haplotypes were found for *S. acus*, but also common sequence variants respect to Northern and Southern European populations of *S. acus* (1 haplotype) and *H. guttulatus (3 haplotypes)*, respectively [100, 101]. Available genetic sampling in PNAC allowed detecting novel haplotypes for a small number of pipefish morphologically identified as *N. maculatus* (one 16S rDNA haplotype in a single specimen) and *S. abaster* (two Cytb haplotypes for six individuals). These two new *S. abaster* Cytb haplotypes detected in PNAC clustered in a single monophyletic group, supporting the morphological identification, but separately from previous Cytb sequences available for voucher samples of the same species from Italian coasts [58], and also from other congeneric pipefish distributed in the Mediterranean Sea (*S. acus*, *S. rostellatus*, *S. taenionotus*, *S. typhle*). Morphological discrimination from other possible species like *S. schmidti* and *S. phlegon* was also stablished based on non-overlapping ranges for meristic traits (http://species-identification.org/index.php,[37], in the absence of available Cytb data to be compared in these species. Some sample misidentification during in situ surveys could be possible, according to confuse discriminations previously reported for some Mediterranean *Syngnathus* species, like *S. rostellatus* [37]. However, the combined morphological and genetic results in our study are congruent with previous data based on different mitochondrial markers, which support that *S. abaster* does not constitute a monophyletic taxon [81]. Indeed, highly divergent *S. abaster* mitochondrial lineages were described in the westernmost Mediterranean Sea respect to more eastern Italian coasts, which may be acknowledged as distinct related species [81]. The Cytb haplotypes in this study confirmed a strong differentiation between the Italian *S. abaster* lineage, represented by reference samples reported by Mwale et al. [58] and the *S. abaster* haplotypes from PNAC, in Balearic Islands, which has been proposed to be part of the westernmost group along with adjacent sectors in Sardinian Sea [81]. Genetically divergent populations in species associated with long term isolation and restricted potential for dispersal has been pointed in different species inhabiting marine coastal habitats, including syngnathids, such as reported for the north-western Pacific messmate pipefish [81, 86].

Differences in diversity, distribution and abundance of syngnathids are related to habitat characteristics [62, 96, 101]. Many species are algae and seagrass residents closely associated with specific habitats that best enable camouflage [26, 45, 54, 82, 102]. In PNIA, seaweed communities are structurally complex and patchily distributed on mixed or rocky substrates [66, 69]. Most syngnathids in PNIA were located in semi-exposed or sheltered habitats on areas that showed the highest similarity regarding seaweed communities. Those areas were clearly identified in the estimated distribution map, and include rocky and sandy-gravel substrates, maerl beds as well as seaweed communities enhancing protection and habitat suitability for syngnathids. Transect TR10 was particularly interesting since it was located in the most sheltered area and the unique site with seahorse occurrences. As for *S. acus* in PNIA, dominant pipefish species form monospecific populations [54, 96] but many European pipefish species may vary their habitat occupancy and overlap a great deal [96], as shown in PNAC. Seagrass meadows are lacking in PNIA [29, 30] but PNAC seabed was partially covered by large extensions of seagrass meadows (*P. oceanica* and *C. nodosa*), which is a typical cover enhancing the occurrence of syngnathids in some Mediterranean areas [99]. That is the case of *S. typhle*, a pipefish that preferentially displays an upright position in seagrasses with narrow leaves (e.g. *Zostera*) [84, 96]. Its absence in PNIA could rely on the lack of seagrass meadows, even though this species may adapt to different types of habitats [88]. Appropriate habitats for syngnathids may not be determined simply by the presence or absence of vegetation but also by the prevalence of seaweed communities that best enable them to remain inconspicuous to predators [45]. All pipefish in PNAC were collected in *C. nodosa* and *P. oceanica* meadows, suggesting that macroalgal beds are less preferred than seagrass meadows.

In PNAC, the results showed unexpected low pipefish abundance, which agrees with previous observations in similar habitats [96]. The highest abundance was recorded in *C. nodosa* meadows in Es Burri Bay (1.2–1.3 syngnathids 100 m^−2^). Visual censuses of syngnathids in dense meadows are difficult due to fish crypsis. Captures with the first visual censuses from 2.8 to 21.5 m depth resulted substantially improved with *gánguil* gear operating at 11–16.5 m depth. However, global species richness and abundance in PNAC could have been underestimated. European syngnathids usually inhabit brackish areas (< 10 m depth) but *C. nodosa* meadows are present at deeper depths (11–13 m depth) in Es Burri Bay. The dominant pipefish *S. abaster* in PNAC commonly inhabits at 0.5–5 m depth [20, 21], which is clearly above the depths imposed by gear, site and fishing permissions in *gánguil* sampling.

Changes in macroalgal assemblages in PNIA are occurring since 2012. Abundance of *Treptacantha baccata, T. usneoides* and *Saccorhiza polyschides* decrease, while turf (*Halopteris scoparia*, *Chondria coerulescens* or *Corallina* spp.), and non-native (*Codium fragile*, *Asparagopsis armata*) species increase [12]. The progressive habitat loss and the increase in less optimal seaweed species can also cause dramatic changes in resident fauna and community composition [89, 90]. Most syngnathids from PNIA were captured in shallow waters (< 10 m depth) on sandy substrates with low proportions of gravel, some mud and preferably nearby rocky outcrops that provides better refuge and protection (TR3, TR10). Coastal sheltered areas protected from SW (TR1) and N waves (TR7–TR9) were preferred but areas with high bottom mobility (sand waves and megarriples 3D) were avoided (TR2 and TR4). Some syngnathid species appear to be generalist considering distribution patterns and algal community characteristics whereas others prefer certain seaweed forms and feed on specific sources [54, 71]. Distribution patterns can be partially explained by the exposure to waves and open sea [54, 85] which has a great impact on seaweed cover. However, *S. acus* was also able to inhabit shallow and rocky areas (TR3) near the shore wave-breaking zone submitted to a certain degree of water agitation.

Due to the high dominance of *S. acus* in PNIA, the species deserves special consideration. The length–weight relationship was similar to that in the western Black Sea [105]. The large specimens (31.8 ± 10.0 cm SL) in PNIA was noteworthy compared to PNAC and other Mediterranean populations but did not differ from others in eelgrass meadows from Northern Europe [20, 35, 36, 96, 105]. However, the absence of small-medium sized specimens in Cíes Archipelago raises the questions of whether there is a resident population of adults (with dispersal of small individuals towards other areas) and/or whether the fishes migrate seasonally to Cíes from nearby areas only for breeding. None of the specimens marked in spring were recaptured in summer, suggesting that they might not be so site faithful as reported [96]. A comparative isotopic study and further genetic analysis with informative markers representative of specimens from nearby areas would clarify that dilemma.

### Breeding season

Syngnathids may change habitat and prey preferences as they grow [21, 29, 47, 64]. The absence of small and medium-sized immature specimens in Cíes suggests that young fishes prefer less exposed nearby sites, and/or that small juveniles are dispersed by currents to other areas. In *S. acus*, sexual maturity in the Aegean Sea is reached at lengths of 7.7 cm in females and 8.1 cm in males [36]. The smallest *S. acus* captured in PNIA measured 14.8 cm TL.

In our study, mature specimens of *S. acus* were present in early May–June but not in early September. These findings agree with the reported breeding season for the species (January to August), with peaks of hydrated-oocyte carrying females and pregnant males in March–July depending on latitude and temperature [5, 38, 100]. In Cíes Archipelago, temperatures raised from 14.1 in May to 18.1 °C in June, and dropped to 16.3 °C in September. Hence, the breeding season in syngnathids from PNIA seems to be limited by water temperature [56]. In PNAC, the small number of pipefish and their small size prevents from concluding anything on this topic.

### Isotopic signatures in syngnathids

The extreme scarcity of pipefish in PNAC prevents from concluding remarks on isotopic patterns and trophic characteristics of pipefish. Pipefish from PNAC showed lower isotopic signals (particularly for δ^15^N) than in PNIA, which agrees with some isotopic values in Mediterranean zooplankton [80] but not with those in other Mediterranean areas [99]. Disagreements might be driven by differences in resource exploitation and resource partitioning (especially organic matter sources at the base of the food web) depending on the study site.

**Fig. 4:**
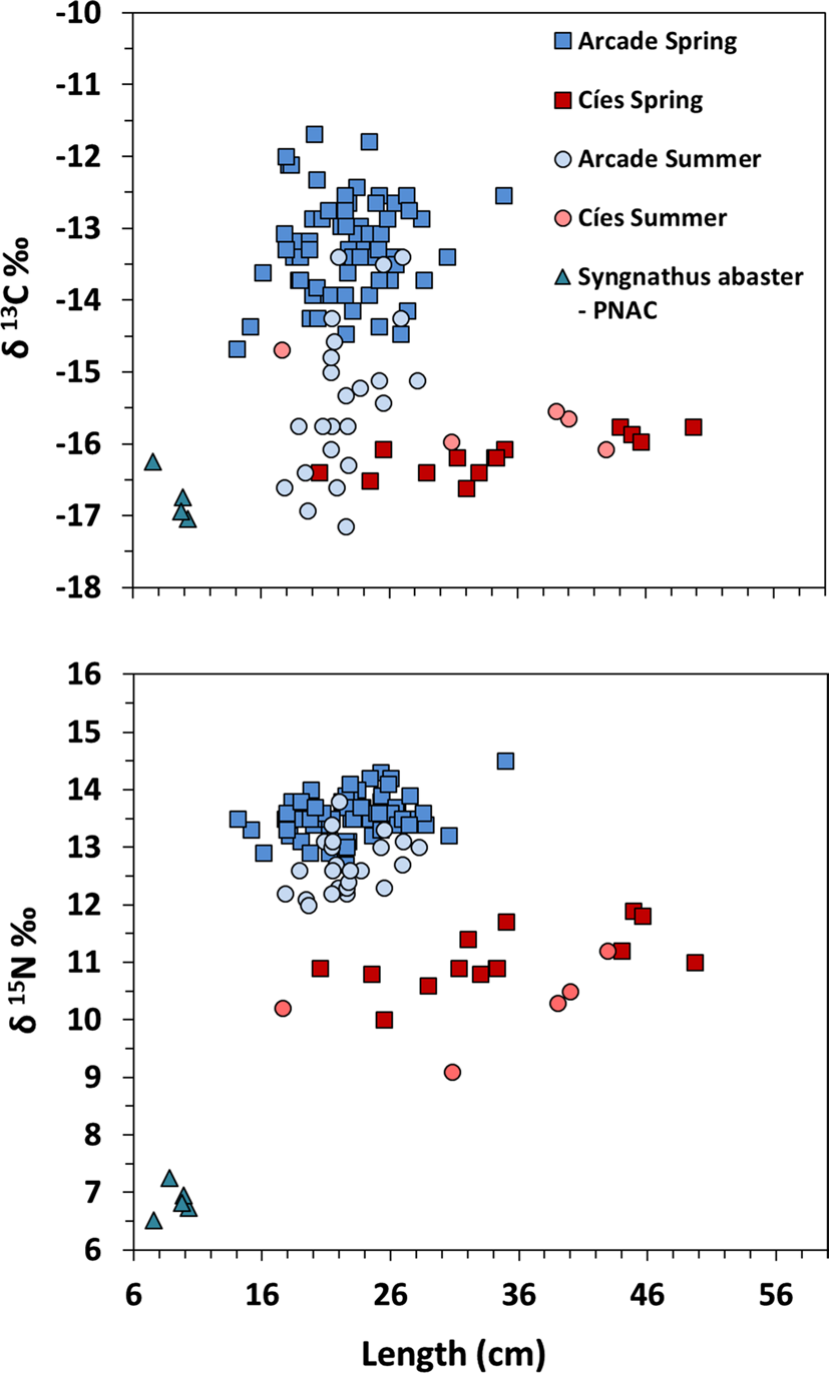
Scatter plot of stable isotopes-length relationships in *Syngnathus acus* captured in spring and summer 2016 in Cíes Archipelago (PNIA). Data for Arcade Cove pipefish (M. Planas, unpublished observations) and for *S. abaster* from PNAC (December 2016) are provided for comparative purposes. Arcade Cove specimens were collected on spring and summer 2016

**Fig. 5:**
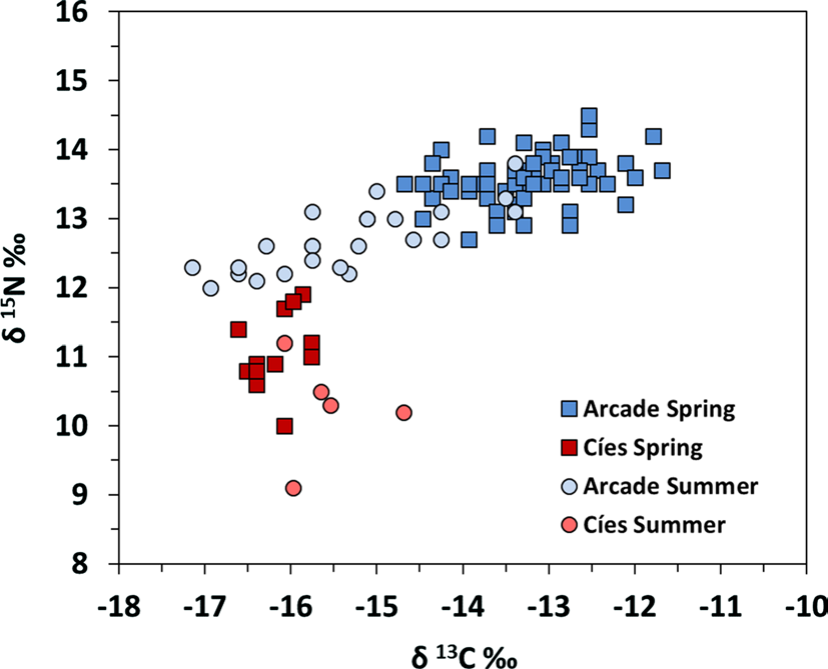
Schematic representation of the relationships between isotopic values (δ^13^C and δ^15^N and *Syngnathus acus* from Cíes Archipelago (present study) and Arcade Cove (M. Planas, unpublished observations). Samples collected in spring and summer 2016. Similarity groups (polygons) from hierarchical clustering (Ward's method) are shown

The pelagic food web from coastal areas (e.g., Arcade cove) in Galicia are typically enriched in δ^13^C and δ^15^N compared to more oceanic areas (e.g., Cíes Archipelago) [8]. Arcade cove is located in San Simón Bay on the inner part of Ría de Vigo (30 km from Cíes Archipelago). The cove is a shallow mesotidal *Zostera* meadow with low hydrodynamic conditions but receiving freshwater inputs [2]. The population of *S. acus* inhabiting Arcade cove markedly differs in size and isotopic signals from that in Cíes (Fig. 4). Habitat and trophic web characteristics in both areas also differ considerably [24]. The former receives anthropogenic wastewater inputs, being characterized by a complex trophic web, and a locally important microphytobenthos production available to primary consumers through resuspension. The cove is a community with a high diversity of organic matter sources but terrestrial particulate organic matter does not seem to contribute significantly to consumers’ most plausible diets [24]. Wastewater discharges would increase δ^15^N values in Arcade cove as shown in other similar areas [8]. Surface dissolved nitrogen concentrations (DIN) and isotopic discrimination for δ^15^N in Ría de Vigo are typically higher from October to April, decreasing from May to September [60]. Hence, higher δ^15^N signatures in spring–summer would be expected [78, 79]. The opposite trend would occur in δ^13^C signatures [79]. Both trends were reflected in isotopic signals of *S. acus* in Arcade cove (Fig. 5) but not in those from Cíes, with higher oceanic influence and more stable conditions. The higher δ^15^N signatures in Arcade cove were reflected in isotopic signatures of the whole web trophic chain [24], including *S. acus* specimens (13.3 ± 0.5 ‰; range: 12.0–14.3 ‰) [71] (Fig. 5). Considering a trophic enrichment factor of 4.1 for δ^15^N [71] and δ^15^N values of filter-feeders in Arcade cove (8.98 ‰ in *Mytilus galloprovincialis*) [24] and in Cíes (5.13 ‰ in *Musculus costulatus*) [44] as isotopic baselines [73], the resulting trophic levels for *S. acus* in Arcade and Cíes were 3.16 and 3.83, respectively. Such trophic level dissimilarities imply differences in resources exploitation as the result of disparities in trophic web composition and structure.

### Hypothesizing migratory events in PNIA

Isotopic profiles in tissues has proven useful to build isoscapes and infer geographic origins and spatial connections [41, 104], which was not the scope of our study. However, some hypothesis can be provided on potential migrations in *S. acus* on Cíes Archipelago. Isotopic signals in fin tissues of syngnathids reflect the isotopic profile of the diet ingested 2–3 months earlier (M. Planas, unpublished observations). Assuming the existence of winter-spring migratory events from areas nearby Cíes, fin isotopic signals in pipefish captured in Cíes in summer would reflect those of the diet ingested on a nearby area in spring. However, that assumption is not supported by actual differences in δ^15^N signatures (2.7 ‰) between specimens from Arcade in spring (13.8 ± 0.4 ‰; range: 13.2–14.5) and those from Cíes in summer (10.1 ± 1.5 ‰) (Fig. 5). The sharp drop in abundances from late summer [44] and in the absence of more data, we hypothesize that spring–summer populations of *S. acus* in Cíes Archipelago are mainly founded by large resident specimens, undergoing migration into adjacent habitats to avoid harsh autumn–winter conditions as reported in other syngnathids [26, 49, 54, 56, 96]. Subsequently, the species would return to Cíes in early spring for breeding when males begin developing their brood structures and after plant-cover recovery.

### Directions for research and conservation

There is increasing public awareness of the challenges of marine biodiversity from habitat destruction, over-fishing and development. Efforts are needed to protect and value marine biodiversity, especially species and communities that require relatively large areas of undisturbed habitat. NPs are areas set aside for the preservation of the natural environment to protect natural biodiversity along with its underlying ecological structure and supporting environmental processes, and to promote education and recreation (IUCN).

A lack of information on syngnathid populations has prevented conservation actions from being conducted in Spanish NPs. Further studies should provide more knowledge on those populations in order to undertake specific conservation actions PNIA and PNAC differ in regulatory and environmental protections, and biota and abiotic components, which determine population characteristics of inhabiting syngnathids. Both high quality environments are tourist destinations supporting at least one jetty, bollards where ships can tie up under permission, and an internationally recognized wildlife. Due to the lack of previous studies, the trend of syngnathid populations is unknown and their future is uncertain. The main concern is the human and fishing pressure, particularly in PNIA. Current diversity and abundances of syngnathids in marine Spanish NPs are extremely scarce, with only five species identified, and there is the need of protecting those limited populations. Our recommendations of potential management and research priorities are as follows:

- The present study provided first data on syngnathid populations in Spanish NPs. The availability of historical data and a continuous monitoring of syngnathid populations and temporal-seasonal variability are imperative for trends assessment. Hence, an objective of this study was the selection of specific sites for further monitoring (Additional file 1). The higher proportion of species/abundances and the distribution map (Maxent) in PNIA indicate that further monitoring should focus on south-eastern-PNIA (TR3 and TR4TR5) and particularly in Rodas Bay (TR10). Rodas Bay is also interesting from a conservation point of view since it is also a preferential habitat for small *Octopus vulgaris* [32]. In PNAC, considering the benthic communities and the higher abundance of pipefish compared to other locations, Es Burri Bay has the greatest interest value for further conservation actions and monitoring. Studies not based on long-term monitoring may lead to erroneous or incomplete assumptions. The limited captures of fishes along the present study and the lack of data on pivotal subjects such as bionomic data or connectivity between potential analysis units impeded the use of conservation planning tools. However, it is expected that further monitoring will provide the necessary captures and data to allow more accurate distribution maps for syngnathids in both NPs and the preparation of a reserve design in order to reprioritize the areas for conservation and management actions, zoning multiple-use and minimising conflict of use. The future study will provide valuable information on the assessment of species’ sensitivity to habitat disturbances and climate warming [23], and on optimal conditions for captive breeding and further population reinforcement for the most endangered species if necessary.
- Some of the main questions arisen from the study is whether Cíes Archipelago should be considered a breeding sanctuary for *S. acus*. Understanding fish movement patterns and migrations from/to other nearby areas is another pivotal topic than needs addressing. For that, further isotopic and genetic information, and acoustic telemetric studies in specimens tagged with transmitters would provide valuable information to undertake further conservation actions [95].
- The management of vessel transits to protect sensible areas against habitat loss (marine flora communities) and to mitigate anthropogenic sound is necessary [55]. Long-term soundscape monitoring and more restricted vessel anchorage conditions for resource management [40] are recommended in Rodas Bay (TR10 in PNIA), which supports a high density of vessel traffic during the touristic seasons. Seagrass meadows appear as essential communities to maintain syngnathid populations in PNAC as all fishes were captured there. Damage to seagrass meadows by anchoring of recreational boats in Es Burri Bay might compromise syngnathid populations in PNAC.

## Conclusions

This is the first multidisciplinary study of syngnathid populations in Spanish coasts, specifically in the two marine Spanish NPs. It will contribute to the knowledge of syngnathid populations, leading to more informed and efficient management of both NPs. Species diversity, abundance, habitat preference, and isotopic signatures differed in both NPs, depending on habitat characteristics. Syngnathids preferred sheltered macroalgal assemblages in PNIA and *Cymodocea* meadows in PNAC. Our results seem to indicate that PNIA is a breeding sanctuary for *S. acus*, which migrate seasonally. Genetic markers agreed with meristic characteristics, except for *S. abaster* in PNAC, suggesting the presence of a divergent mitochondrial lineage within a polyphyletic *S. abaster* species complex, and the need of further genetic and morphological research to clarify its taxonomic status respect to other Mediterranean populations and the conservation consequences. Preferential sites for future monitoring of syngnathid populations in both NPs, some actions to undertake for conservation purposes and further research priorities are proposed. Syngnathids, particularly seahorses, are flagship species attracting the attention of citizens. Efficient further actions will enhance public engagement with marine biodiversity, resulting also in social, economic and wellbeing profits.

## Methods

### Study areas

**Fig. 6:**
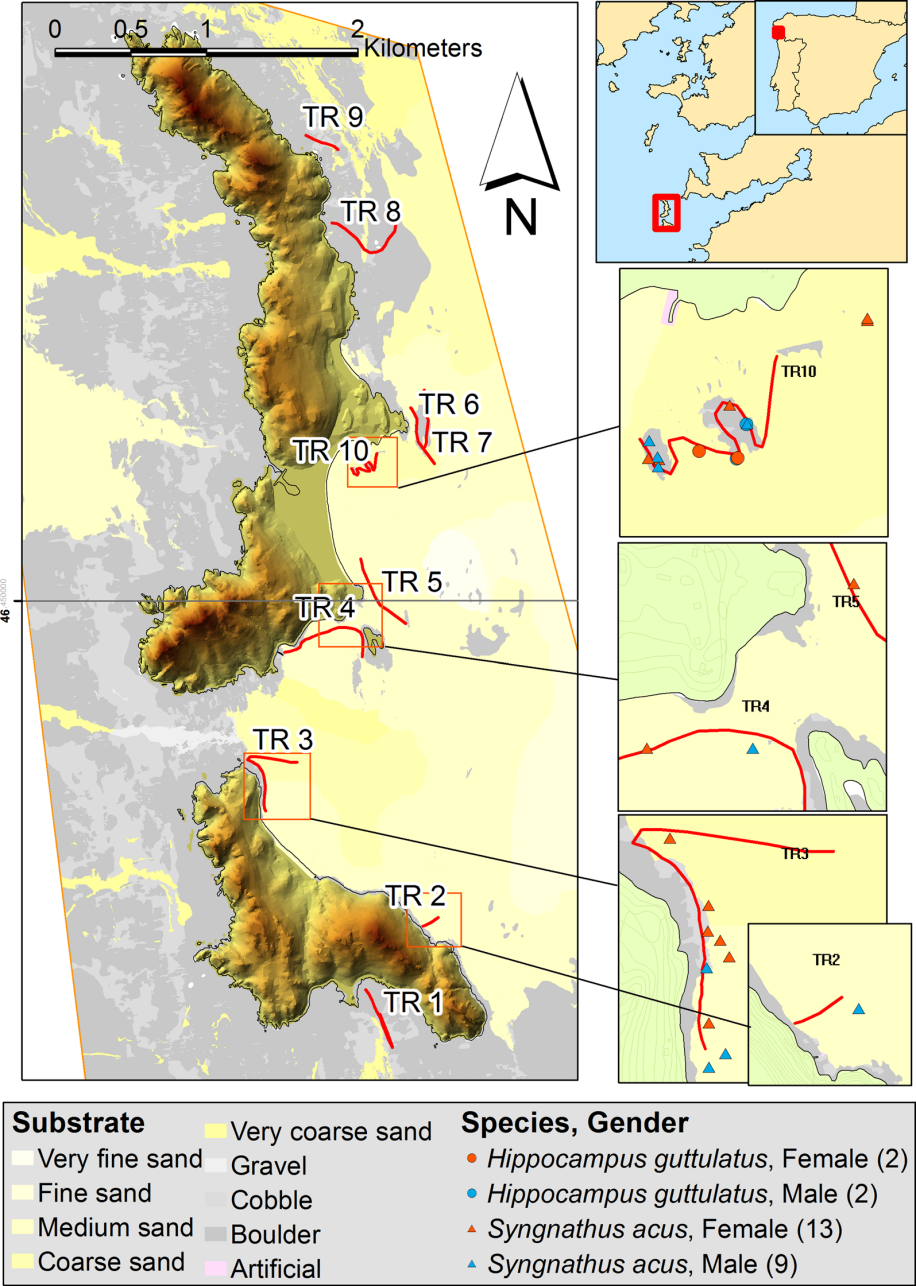
PNIA—Study area and transects (red lines; TR1 to TR10) surveyed for syngnathids in Cíes Archipelago (Galicia, NW Iberian Peninsula). Transects TR2-TR5 included rocky outcrops and sandy substrates, but resolution in the map at the presented scale do not show the rocky outcrops. We acknowledge the information provided by OAPN on GIS layers

**Fig. 7:**
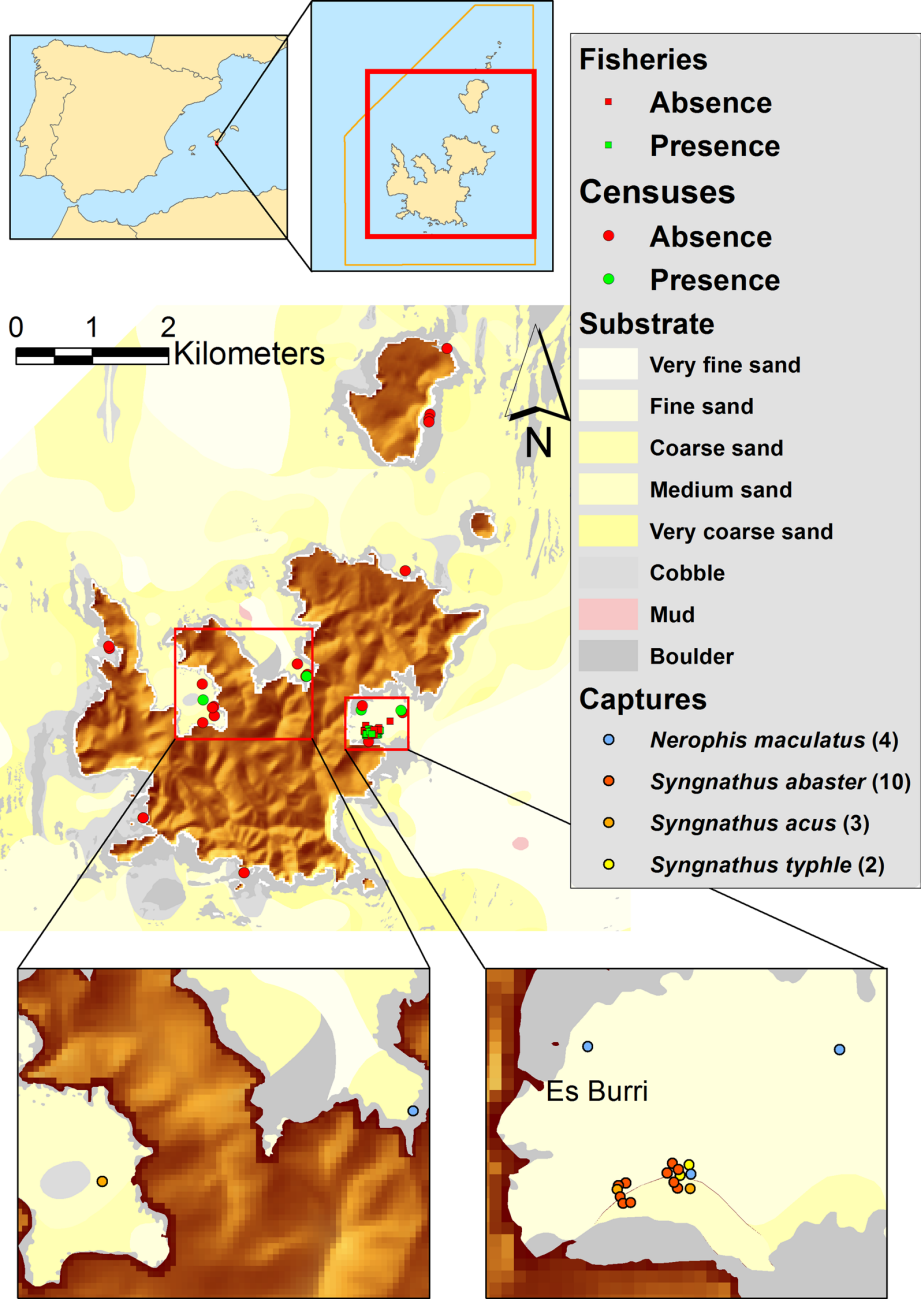
PNAC—Study area and surveyed sites for syngnathids in Cabrera Archipelago (Balearic Islands, West Mediterranean). Upper: Presences and absences of syngnathids. Below: Capture sites in Cabrera Island. We acknowledge the information provided by OAPN on GIS layers

The study was carried out in (a) Cíes Archipelago (42°13′ N, 8°54′ W), in Atlantic Islands National Park (PNIA), located on the outer area of the Ría de Vigo (NW Iberian Peninsula) (Fig. 6), and (b) Cabrera Archipelago National Park (PNAC) (39°08′ N, 2°56′ W), in the western Mediterranean (Balearic Islands) (Fig. 7).

The study in PNIA was conducted in Cíes Archipelago, comprising three islands and various islets. PNIA was declared Nature Reserve in 1980 and Spanish National Park in 2002. The NP is located at the northern limit of the eastern boundary upwelling system off NW Africa and SW Europe. Northerly winds induce coastal upwelling in this region during most of spring and summer [28] and colder nutrient-rich subsurface water known as Eastern North Atlantic Central Water (ENACW) inside the estuaries [1, 60]. Cíes Archipelago was declared Natural Park, Special Protection Area (SPA), Site of Community Importance (SCI), OSPAR area, and UNESCO World Heritage candidate [91]. Seawater temperature is homogeneous in winter (13–16 °C) and stratified in summer (12–18 °C) due to the warming of upper layers. Surface water temperature typically ranges from 13.4 to 18.7 °C in the southern coast and from 13.4 to 18.0 °C in the northern coast [75].

In PNAC, oceanographic data indicate stratification of summer water column, horizontal distribution of water masses and hydrodynamic features linked with Mediterranean seasonality [14]. Waters up to 100–150 m depth are highly influenced by Atlantic Ocean water entering the Mediterranean through the Gibraltar Strait. Sea surface temperature ranges between 14.6 °C in winter and 27.5 °C in summer [6]. Coastal waters are oligotrophic due to low concentrations of dissolved inorganic nutrients and chlorophyll [98] and light attenuation coefficient is extremely low. Depth and hydrodynamics are the dominant abiotic factors that affect habitat distribution and vary among sites throughout the archipelago [6].

### Swept sites

Based on previous knowledge (seaweed cover, substrate characteristics and exposure level to open water), ten subtidal transects (TR1 to TR10) were selected along the western coast of Cíes Archipelago in PNIA (Fig. 6; Additional file 1), and visited in spring and summer 2016 (two visual censuses per site and season) to obtain an overview of habitat characteristics and spatial distribution of syngnathids. Transects were positioned parallel or perpendicular to the coastline (150 to 700 m length; 3–20 m depth) on rocky bottoms often interrupted by sandy patches. Two pairs of divers conducted 40 diurnal standard underwater visual census (UVC) (50 min per survey; 160 diving hours) along the East coast, covering a total surface of 8.22 ha (10 transects, 5 m wide). All syngnathids sighted were recorded and captured by the divers searching adjacent (belt transects) and separated by the maximum distance allowed for horizontal visibility (commonly 2.5 m). One pair of divers also recorded the characteristics (species, seaweed cover) of seaweed communities, and the other pair sampled the sediment.

Average temperatures were calculated using data of the Galician Oceanographic Network (MeteoGalicia database; www.meteogalicia.gal) from a buoy located in the southern area of Cíes Islands (42°10.691′ N, 8°53′589 W), recording average daily temperatures at 6 m depth. Survey water temperatures were calculated as the average temperature for the period comprising one week before and after the sampling day.

Soft bottom substrates were found among rocky outcrops or in the edge of rocky reefs. To characterize sediments of the swept area in PNIA, the uppermost 2 cm of sediment were underwater manually collected using plastic pots along each transect and considering changes in bottom characteristics. Through and crest zones were also sampled when bedforms were present. A total of 76 sediment samples was collected in spring (52) and summer (24), and conserved at 4 °C for further textural and compositional analysis.

PNAC includes a main island (Cabrera) and a group of four minor islands and several islets. Eleven subtidal sites (TR1 to TR11) including the main shallow benthic habitats present in PNAC were visited from 21st April to 1st December 2016 (17–26 °C) throughout the coast of Cabrera and Conillera islands (Fig. 7) for an overview of syngnathids distribution. We conducted 37 surveys using UVC (50 m length × 5 m wide; 60–80 min per dive; at least two visual censuses per site). A total surface of 0.925 ha was surveyed covering a depth gradient from 2.8 to 21.5 m. UVC were performed on *Posidonia oceanica* meadows, *Cymodocea nodosa* meadows, photophilic macroalgal beds on rocky substratum and mixed habitats formed by these communities. Two pairs of divers participated in each survey, recording and capturing all syngnathids sighted. Depth, water temperature, position and habitat type (substrate, benthic community) were annotated for each fish captured.

Due to the low number of syngnathids encountered with UVC in PNAC, a small trawl net called *gánguil* (traditional gear for small crustaceans catching) was assayed on *C. nodosa* meadows. The gear has a rolling stainless steel cylinder incorporated in the bottom of the mouth for protecting *P. oceanica* and *C. nodosa* leaves from snagging and tearing while operating. The beam trawl was 3 m long and it had a 0.8 m mouth aperture with 1.2 cm^2^ mesh size [15]. To avoid damage of fan mussel, *Pinna nobilis*, populations while sampling, PNAC authority only allowed the use of *gánguil* in *C. nodosa* meadows in Es Burri Bay (Fig. 7; 39°8.604′ N 2°57.524′ E). Seven fishing sets were carried out from September to December 2016, covering a total area of 0.114 ha from 11 to 16.5 m depth.

The depth, position and habitat type (also substrate and seaweed assemblages in PNIA) were annotated for each fish captured. Flora and fauna nomenclature followed codes of Guiry and Guiry [34] and WoRMS Editorial Board [103]. Swept areas were calculated according to Guerra et al. [33], considering the effective sampling time, the net sampling distance, the distance between divers and the number of divers.

### Fish collection

In UVC, syngnathids were hand-caught collected or manually extracted from the fishing gear, introduced in numbered plastic bags and transferred to a support boat. In PNIA, once on land, the fish were morphologically identified, anesthetized with Ethyl 3-aminobenzoate methane sulfonate (MS-222; 0.1 g L^−1^; Sigma-Aldrich Co., USA) and marked subcutaneously using visible implant fluorescent elastomers (VIFE; Northwest Marine Technology Inc., USA) on the ventral surface of the trunk (pipefish) or laterally (seahorses). All anaesthetized fish were weighted (W, g) and sized for standard length (SL, cm). In PNAC, the fish were morphologically identified on board, anesthetized, sized as reported above but not weighted because it was not possible to stabilize the balance in boat conditions. A fraction of the fish collected by fishing in PNAC were sacrificed for sampling (stable isotopes and genetic analysis) due to their small size (with permission of NP authority).

Dorsal fin samples were taken by fin- clipping [70], transferred to screw-capped tubes containing 95% ethanol and conserved at 4 °C for further genetic and stable isotope analysis (SIA). The presence of previous marks (recapture events), sex, sexual status, meristics (fin rays, body rings) and body coloration were also annotated whenever possible. The sexual status was recorded considering pregnancy in males and trunk shape (holding of hydrated eggs) in females. Species identification was evaluated genetically using DNA extracted from dorsal fin samples available from PNIA and PNAC surveys. In PNIA, all fishes from visual censuses were released at the capture site within 2–3 h after sampling.

For SL measurement, the fishes were placed on a plate including a measurement scale and photographed laterally (seahorses) or measured directly (pipefish). Seahorse images were analysed in the laboratory to determine length using image-processing software (NIS Elements Nikon and ImageJ2). Seahorses were measured as head + trunk + tail length (curved measurement in seahorses) [52].

Allometry in fishes was assessed using the following equation:

$${\text{TL}} = {\text{aW}}^{{\text{b}}}$$

where, TL is total length, a is an empirical coefficient, W is body weight and b is the allometric exponent.

### Sediment analysis

The analysis of sediments was only carried out in PNIA. For compositional analysis, the content of organic carbon and inorganic carbon (calcium carbonate content is equivalent to bioclastic component for this regional setting) was determined by a LECO CNS-2000 Macro Elemental Analyser at CACTI (University of Vigo). Those analyses were performed on the fractions < 2 mm, in order to avoid distortional results due to gravel components (> 2 mm, maerl, bivalve and gastropod shells).

For textural analysis, the bulk grain size distribution was performed by dry sieving. Previously to grain size analysis, the organic matter was removed using 30% H_2_O_2_ for several days and salts were removed with further washings with distilled water. Afterwards, the samples were dried at 50 °C and dry sieved between 4 mm and 63 μm (sieve size intervals of 1/2 ø). The resulting grain size distribution was treated with the GRADISTAT program [7]. For statistical parameters (mean, selection, asymmetry and kurtosis or pointing of the grain size curve), the nomenclature of Folk and Ward [25] classification was used.

### DNA sequence analysis

DNA was extracted from dorsal fin tissue collected from the following morphologically identified specimens: (i) twenty-two wild greater pipefish (*Syngnathus acus*) and four long-snouted seahorses (*Hippocampus guttulatus*) from PNIA; and (ii) six black-striped pipefish (*S. abaster*) and one spotted pipefish (*Nerophis maculatus*) from PNAC. Genomic DNA was isolated using NucleoSpin Tissue XS kit (Macherey–Nagel Inc., Germany) and for extremely small tissue samples further amplified using GenomiPhi V2 kit (Healthcare, USA).

The mitochondrial marker cytochrome b (Cytb) used for phylogenetic analysis in the Family Syngnathidae [100] was assayed for the molecular identification of all specimens studied from two divergent phylogenetic groups (Syngnathinae and Nerophinae subfamilies,[38]. Universal primers L14275F [64] and H15926R [100] were used to amplify Cytb in the pipefish species, while the specific primers SHORSE5.3L [13] and GUTCYTBR [101] in seahorses. To overcome the poor Cytb amplification success in *N. maculatus*, the universal primers 16Sa-L2510 and 16Sb-H3080 [65] were used in this species to amplify 16S rDNA, an informative marker also used for phylogenetic analyses in Syngnathidae [100]. PCR reactions in 50 μL included 100 ng of template DNA, 1X PCR Gold Buffer (Applied Biosystems), 2.5 mM of MgCl_2_, 400 µM of dNTPs, 0.2 µM of each primer and 1 and 1.25 units of Amplitaq Gold™ DNA polymerase (Applied Biosystems) for pipefish and seahorse, respectively. Specific PCR programs were used for pipefish (95 °C for 10 min, 33 cycles of 93 °C for 1 min, 50 °C for 1 min and 72 °C for 3 min, plus final extension at 72 °C for 10 min) and seahorses (94 °C for 10 min, 35 cycles of 94 °C for 30 s, 50 °C for 30 s and 72 °C for 1 min, plus final extension at 72 °C for 2 min). Sequences were obtained using the ABI PRISM BigDye™ Terminator v3.1 Cycle Sequencing Kit on an ABI PRISM® 3730xl Genetic Analyzer (Applied Biosystems, Foster City, CA). Variable sites were checked with SEQSCAPE 2.5 (Applied Biosystems), using Genbank sequences AF356040, AF354994 (from Sweden; [100] and AF192664 (from UK [13], as reference for *S. acus*, *N. ophidion* and *H. guttulatus*, respectively. Variable positions, haplotypes and genetic distances (estimated with the p-distance method) were obtained using MEGA 7.0 [48] while haplotype diversity [59] for the different species was calculated using DnaSP 5.0 [50]. Species identification of sampled haplotypes was performed using BLASTn tool with default parameters within NCBI database. Evolutionary relationships among *S. abaster* haplotypes from PNAC and GenBank sequences of Mediterranean-distributed *Syngnathus* species were inferred using the Neighbor-Joining method based on p-distance implemented in MEGA with *S. exilis* (JF273424) as outgroup [58], and clustering support evaluated using bootstrap test (1000 replicates).

### Stable isotopes analysis (SIA)

For δ^13^C and δ^15^N analysis in syngnathids, the samples were rinsed with distilled water, transferred to tin capsules, dried in oven at 60 °C for 24 h and weighted (± 1 μg). Due to the low lipid content in fin samples conserved in ethanol (< 5% lipids, C/N < 3.56) [74], further full defatting was not necessary [72, 93]. Samples were analysed at SAI (University of A Coruña) by continuous flow isotope ratio mass spectrometry using a FlashEA1112 elemental analyser (Thermo Finnigan, Italy) coupled to a Delta Plus mass spectrometer (FinniganMat, Germany) through a Conflo II interface. Isotopic values are expressed as permil (‰) in conventional delta relative to VPDB (Vienna Pee Dee Belemnite) and Atmospheric Air. The precision (standard deviation) for SIA of the laboratory standard (acetanilide) was ± 0.15‰ (1-sigma, n = 10).

### Geographic information

GIS was managed with ArcGIS v.10.5 software to represent the maps. Layers of bionomic maps for both NPs (OAPN, unpublished observations) were incorporated. Sampled sites/transects and syngnathid capture locations were recorded and added to a geodatabase. Biological information of the specimens (species, sex, size, weight and sexual stage) was joined to each register. Available abiotic information (topographic and bathymetric layers), as well as bionomic information, were also added to geodatabase. Cartographic data were projected in UTM 29 N/UTM 31 N reference system (for PNIA and PNAC, respectively) using ETRS89 Datum.

### Species distribution estimates

Modeling distribution of syngnathids was only assessed in PNIA as the number of specimens collected in PNAC was insufficient. For that, Maxent (Maximum Entropy model) was implemented [67, 68], [24], using MaxEnt v.3.4.1 program (https://biodiversityinformatics.amnh.org/open_source/maxent/). For modelling, bathymetric, substrate and oceanographic variables were used as predictors of species habitat suitability. Bathymetry (BM) and seabed slope were used as bathymetric variables. BM data were obtained from the PNIA cartographic database (PNIACD; unpublished), which was provided for the managers of PNIA. Slope was derived from the bathymetric layer using the Spatial Analyst tool from ArcMap (ArcGis 10.5). Slope describes the proportion of change in elevation over distance. Low values of slope are associated with flat sea bottoms, while higher values indicate potential rocky bottoms. Sediment Texture (ST) was used as substrate variable and it was introduced in the models to define the sediment substrata. ST data were obtained from PNIA cartographic database. The ST map was constructed according to the Krumbein’s Phi Scale [46], using the following ST classes as a function of the diameter of the particle: very fine sand, fine sand, medium sand, coarse sand, very coarse sand, gravel, cobble and boulder. Waves Exposure (WE) was used as oceanographic variable. WE values were extracted from the Model of Waves of Galicia (Meteogalicia database, www.meteogalicia.gal). WE describes the annual mean power per meter wave front. Low values of WE are associated with sheltered areas, while higher values suggest high influence to wave force.

### Data analysis

All means are reported with standard deviation. The data were checked for normality and homogeneity of variances (Shapiro–Wilk and Levene’s tests). Analyses of variance (ANOVA) were used to examine the effects of season, sex, reproductive status, length, weight and isotopic values in syngnathids. Tukey's HSD test adjusted for unequal sample sizes were performed for post hoc comparisons [87]. Statistical analyses were performed using R packages, with significance set at P = 0.05.

Diversity, species richness and total number of species were estimated for seaweed in PNIA. Differences between transects and seasons were analysed using PERMANOVA for each univariate variable. *P*-values were estimated with an asymptotic permutation distribution generated by the Monte Carlo method. PERMANOVA was also used for seaweed assemblage comparisons across transects and seasons using Bray–Curtis pairwise similarities. Patterns in the structure of assemblages were visualized with principal coordinates (PCO) plots of samples and centroids of each combination of Transect × Time in the Bray–Curtis space. Data and statistical analysis were performed with R (Glht and Factoextra packages) and PRIMER-e v6 and PERMANOVA + for PRIMER (Massey University, New Zealand).

## Supplementary Information

**Additional file 1:** 3D video showing surveyed sites and locations of syngnathid captures in Cíes Archipelago (PNIA). The final sites for further monitoring are also shown.

**Additional file 2:** Textural and compositional parameters of sediment (PNIA), general characteristics of macroalgal species and communities (PNIA), haplotypes detected in syngnathids (PNIA and PNAC), PCO Principal coordinates ordination for seaweed assemblages and syngnathids (PNIA), and length–weight relationships in syngnathids (PNIA).

**Additional file 3:** Illustrations of main species (medium–high abundance) of macroalgae and bathymetric zonation of seaweed assemblages in transects surveyed on Cíes Archipelago (PNIA).

## Data Availability

As we are working on a long-term project, the datasets used and analysed during the current study are available from the corresponding author on reasonable request.

## Abbreviations

C: Total carbon
GIS: Geographic information system
OAPN: Organismo Autónomo de Parques Nacionales españoles
IUCN: International Union for the Conservation of Nature and Natural Resources
ANOVA: Analysis of variance
N: Total nitrogen
NCBI: National Center for Biotechnology Information
NP: National Park
PCO: Principal coordinates analysis
PERMANOVA: Permutational analysis of variance
PNAC: Cabrera Archipelago National Park
PNIA: Atlantic Islands National Park
SCUBA: Self-contained underwater breathing apparatus
SD: Standard deviation
SIA: Stable isotope analysis
SL: Standard length
TR: Transect
UTM: Universal Transverse Mercator system
W: Weight
WORMS: World Register of Marine Species

## References

[1] Álvarez I, de Castro M, Gómez-Gesteira M, Prego R (2005). Inter- and intra-annual analysis of the salinity and temperature evolution in the Galician Rías Baixas-ocean boundary (northwest Spain). J Geophys Res.

[2] Álvarez-Iglesias P, Rubio B, Pérez-Arlucea M (2006). Reliability of subtidal sediments as geochemical recorders of pollution inputs: San Simón Bay (Ría de Vigo, NW Spain). Estuar Coast Shelf Sci.

[3] Álvarez-Salgado X, Gago J, Míguez BM, Gil-Coto M, Pérez FF (2000). Surface waters of the NW Iberian margin: upwelling on the shelf versus outwelling of upwelled waters from the Rías Baixas. Estuar Coast Shelf Sci.

[4] Amengual-Ramis JF, Vázquez-Archdale M, Cánovas-Pérez C, Morales-Nin B (2016). The artisanal fishery of the spiny lobster (*Palinurus elephas*) in Cabrera National Park, Spain: comparative study on traditional and modern traps with trammel nets. Fish Res.

[5] Anonymous. Uova, larve e stadi giovanili di teleostei. In: Fauna e flora del Golfo di Napoli. Monografia 38, Puntata 3. Stazione Zoologica di Napoli; 1956. p. 385–1064.

[6] Ballesteros E, El Bentos ZM. El marc físic. In: Moll-CSIC, editors. Història Natural del Arxipèlag de Cabrera. Palma de Mallorca: Societat d’Història Natural de le Balears; 1993. p. 663–86.

[7] Blott SJ, Pye K (2001). Gradistat: A grain size distribution and statistics package for the analysis of unconsolidated sediments. Earth Surf Proc Land.

[8] Bode A, Álvarez-Osorio MT, Varela M (2006). Phytoplankton and macrophyte contributions to littoral food webs in the Galician up welling estimated from stable isotopes. Mar Ecol Prog Ser.

[9] Buttay L, Miranda A, Casas G, González-Quirós R, Nogueira E (2015). Long-term and seasonal zooplankton dynamics in the northwest Iberian shelf and its relationship with meteo-climatic and hydrographic variability. J Plankton Res.

[10] Caldwell IR, Vincent ACJ (2013). A sedentary fish on the move: effects of displacement on long-snouted seahorse (*Hippocampus guttulatus* Cuvier) movement and habitat use. Environ Biol Fishes.

[11] Cambiè G, Ouréns R, Vidal D, Carabel S, Freire J (2012). Economic performance of coastal fisheries in Galicia (NW Spain): case study of the Cíes Islands. Aquat Living Res.

[12] Carrizosa B. Cambios subacuáticos a corto plazo en el Parque Nacional Marítimo Terrestre de las Islas Atlánticas de Galicia: consecuencias para el uso recreativo del paisaje submarino. Algas. Bol Inf Soc Esp Ficol. 2016;84–8.

[13] Casey SP, Hall HJ, Stanley HF, Vincent ACJ (2004). The origin and evolution of seahorses (genus *Hippocampus*): a phylogenetic study using the cytochrome b gene of mitochondrial DNA. Mol Phylo Evol.

[14] Crechriou R, Alemany F, Roussel E, Chassanite A, Marinaro JY, Mader J, Rochel E, Planes S (2010). Fisheries replenishment of early life taxa: potential export of fish eggs and larvae from a temperate marine protected area. Fish Oceanogr..

[15] Catalán I, Dunand A, Álvarez I, Alós J, Colinas N, Nash RDM (2014). An evaluation of sampling methodology for assessing settlement of temperate fish in seagrass meadows. Mediterr Mar Sci.

[16] Correia M, Caldwell R, Koldewey HJ, Andrade JP, Palma J (2015). Seahorse (Hippocampinae) population fluctuations in the Ria Formosa Lagoon, south Portugal. J Fish Biol.

[17] Correia M, Koldewey HJ, Andrade JP, Esteves E, Palma J (2018). Identifying key environmental variables of two seahorse species (*Hippocampus guttulatus* and *Hippocampus hippocampus*) in the Ria Formosa lagoon, South Portugal. Environ Biol Fish.

[18] Curtis JMR, Vincent ACJ (2005). Distribution of sympatric seahorse species along a gradient of habitat complexity in a seagrass-dominated community. Mar Ecol Prog Ser.

[19] Curtis JMR, Vincent ACJ (2006). Life history of an unusual marine fish: survival, growth and movement patterns of *Hippocampus guttulatus*. J Fish Biol.

[20] Dawson CE, Whitehead PJP, Bauchot ML, Hereau JC, Nielsen J, Tortonese E (1986). Syngnathidae. Fishes of the North-eastern Atlantic and the Mediterranean.

[21] De Maya JA, Andreu A, Miñano PA, Verdiell Cubedo D, Egea A, Oliva Paterna FJ, Torralva M. Dinámica espacio-temporal de la familia Syngnathidae en las áreas someras del Mar Menor (SE, Murcia). Actas del III Congreso de la Naturaleza de la Región de Murcia; 2004. p. 125–31.

[22] Elith J, Graham CH, Anderson RP, Dudık M, Ferrier S, Guisan A, Hijmans RJ, Huettmann F, Leathwick JR, Lehmann A, Li J, Lohmann JG, Loiselle BA, Manion G, Moritz C, Nakamura M, Nakazawa Y, Overton JMcCM, Peterson AT, Phillips SJ, Richardson K, Scachetti-Pereira R, Schapire RE, Soberón J, Williams S, Wisz MS, Zimmermann NE (2006). Novel methods improve prediction of species' distributions from occurrence data. Ecography.

[23] Faleiro F, Baptista M, Santos C, Aurélio ML (2015). Seahorses under a changing ocean: the impact of warming and acidification on the behaviour and physiology of a poor-swimming bony-armoured fish. Conserv Physiol..

[24] Filgueira R, Castro BG (2011). Study of the trophic web of San Simon Bay (Ria de Vigo) by using stable isotopes. Cont Shelf Res.

[25] Folk RL, Ward WC (1957). Brazos River bar: a study in the significance of grain size parameters. J Sedi Petrol.

[26] Foster SJ, Vincent ACJ (2004). Life history and ecology of seahorses: implications for conservation and management. J Fish Bio.

[27] Franzoi P, Maccagnani R, Rossi R, Ceccherelli VU (1993). Life cycles and feeding habits of *Syngnathus taenionotus* and *S. abaster* (Pisces, Syngnathidae) in a brackish bay of the Po River Delta (Adriatic Sea). Mar Ecol Prog Ser.

[28] Fraga F, Richards FA (1981). Upwelling off the Galician coast, Northwest Spain. Upwelling ecosystems.

[29] García-Redondo V, Bárbara I, Díaz-Tapia P (2017). Las praderas de *Zostera marina* L. del Parque Nacional Marítimo Terrestre de las Islas Atlánticas de Galicia y territorios adyacentes: Distribución, abundancia y flora asociada. NACC-Bioloxía..

[30] García-Redondo V, Bárbara I, Díaz-Tapia P (2019). *Zostera marina* meadows in the northwestern Spain: distribution, characteristics and anthropogenic pressures. Biodivers Conserv.

[31] Grau AM, Mayol J, Oliver J, Riera F, Riera MI. Llibre vermell dels peixos de les Illes Balears. Conselleria de Medi Ambient, Agricultura i Pesca. Govern Illes Balears; 2015.

[32] Guerra A, Hernández-Urcera J, Garci ME, Sestelo M (2014). Dwellers in dens on sandy bottoms: ecological and behavioural traits of Octopus vulgaris. Sci Mar.

[33] Guerra A, Hernández-Urcera J, Garci ME, Sestelo M (2015). Spawning habitat selection by *Octopus vulgaris*: New insights for a more effective management of this resource. Fish Res.

[34] Guiry MD, Guiry GM. AlgaeBase*.* World-wide electronic publication, National University of Ireland. 2020. https://www.algaebase.org. Accessed 26 Feb 2020.

[35] Gurkan S, Taskavak E (2007). Length-weight relationships for syngnathid fishes of the Aegean Sea, Turkey. Belgian J Zool.

[36] Gurkan S, Taskavak E, Hossucu B (2009). The reproductive biology of the great pipefish *Syngnathus acus* (Family: Syngnathidae) in the Aegean Sea. North-West J Zool.

[37] Hablützel P, Wilson A (2011). Notes on the occurrence of *Syngnathus rostellatus* (Teleostei: Syngnathidae) in the Mediterranean. Mar Biodivers Rec.

[38] Hamilton H, Saarman N, Short G, Sellas AB (2017). Molecular phylogeny and patterns of diversification in syngnathid fishes. Mol Phylogenet Evol.

[39] Harasti D, Martin-Smith K, Gladstone W (2014). Does a no-take marine protected area benefit seahorses?. PLoS ONE.

[40] Haver SM, Fournet MEH, Dziak RP, Gabriele C (2019). Comparing the underwater soundscapes of four US National Parks and marine sanctuaries. Front Mar Sci..

[41] Hobson KA, Barnett-Johnson R, Cerling T, West JB, Bowen GJ, Dawson TE, Tu KP (2010). Using isoscapes to track animal migration. Isoscapes: understanding movement, pattern, and process on earth through isotope mapping.

[42] IUCN. The IUCN Red List of seahorses and pipefishes in the Mediterranean Sea. 2017. https://www.iucn.org/sites/dev/files/content/documents/2017/ficha_seahorses_baja.pdf. Accessed 6 Nov 2019.

[43] IUCN. The IUCN red list of threatened species. Version 2019‐1. 2019. http://www.iucnredlist.org. Accessed 16 Oct 2019.

[44] Jiménez A. Evolución anual de poblaciones de signátidos del archipiélago de las Islas Cíes (Parque Nacional de las Islas Atlánticas, NO España), Master Thesis Dissertation, University of Vigo; 2019.

[45] Kendrick AJ, Hyndes GA (2003). Patterns in the abundance and size-distribution of syngnathid fishes among habitats in a seagrass-dominated marine environment. Estuar Coast Shelf Sci.

[46] Krumbein W, Sloss L (1963). Stratigraphy and sedimentation.

[47] Kuiter RH (2009). Seahorses and their relatives.

[48] Kumar S, Stecher G, Tamura K (2016). MEGA7: Molecular evolutionary genetics analysis v7.0 for bigger datasets. Mol Biol Evol..

[49] Lazzari MA, Able KW (1990). Northern pipefish, *Syngnathus fuscus*, occurrences over the mid-Atlantic bight continental shelf: evidence of seasonal migration. Environ Biol Fish.

[50] Librado P, Rozas J (2009). DnaSP v5: A software for comprehensive análisis of DNA polymorphism data. Bioinformatics.

[51] López A, Vera M, Otero-Ferrer F (2010). Species identification and genetic structure of threatened seahorses in Gran Canaria Island (Spain) using mitochondrial and microsatellite markers. Conserv Genet.

[52] Lourie S. Measuring seahorses. Project Seahorse Technical Report No.4, Version 1.0. Project Seahorse, Fisheries Centre, University of British Columbia; 2003.

[53] Manning CG, Foster SJ, Vincent ACJ (2019). A review of the diets and feeding behaviours of a family of biologically diverse marine fishes (Family Syngnathidae). Rev Fish Biol Fisheries.

[54] Masonjones HD, Rose E, McRae LB, Dixson DL (2010). An examination of the population dynamics of syngnathid fishes within Tampa Bay, Florida, USA. Curr Zool.

[55] McKenna MF, Gabriele C, Kipple B (2017). Effects of marine vessel management on the underwater acoustic environment of Glacier Bay National Park. AK Ocean Coast Manag.

[56] Monteiro NM, Almada VC, Santos AM, Vieira MN (2001). The breeding ecology of the pipefish *Nerophis lumbriciformis* and its relation to latitude and water temperature. J Mar Biol Assoc UK.

[57] Morales-Nin B, Grau AM, Palmer M (2010). Managing coastal zone fisheries: a Mediterranean case study. Ocean Coast Manag.

[58] Mwale M, Kaiser H, Barker NP, Wilson AB, Teske PR (2013). Identification of a uniquely southern African clade of coastal pipefishes *Syngnathus* spp. J Fish Biol.

[59] Nei M, Tajima F (1981). DNA polymorphism detectable by restriction endonucleases. Genetics.

[60] Nogueira E, Pérez FF, Ríos AF (1997). Seasonal patterns and long-term trends in an estuarine upwelling ecosystem (Ría de Vigo, NW Spain). Estuar Coast Shelf Sci.

[61] Oliveira F, Erzini K, Gonçalves JMS (2007). Feeding habits of the deep-snouted pipefish *Syngnathus typhle* in a temperate coastal lagoon. Estuar Coast Shelf Sci.

[62] Otero-Ferrer F, Herrera R, Tuset VM, Socorro J, Molina L (2015). Spatial and seasonal patterns of European short-snouted seahorse *Hippocampus hippocampus* distribution in island coastal environments. Afr J Mar Sci.

[63] Ouréns R, Cambié G, Freire J (2015). Characterizing the complexity of the fleet dynamics for an effective fisheries management: the case of the Cíes Islands (NW Spain). Sci Mar.

[64] Päabo S, Thomas WK, Whitfield KM, Kumazawa Y, Wilson AC (1991). Rearrangements of mitochondrial transfer-RNA genes in marsupials. J Mol Evol.

[65] Palumbi SR, Martin AP, Romano SL, McMillan WO, Stice L, Grabowski G. The simple fool’s guide to PCR. Department of Zoology, University of Hawaii; 1991.

[66] Peña V, Bárbara I (2016). Los fondos marinos de maërl del Parque Nacional de las Islas Atlánticas (Galicia, España): Distribución, abundancia y flora asociada. NACC (Bioloxía).

[67] Phillips SJ, Anderson RP, Schapire RE (2006). Maximum entropy modeling of species geographic distributions. Ecol Model.

[68] Phillips SJ, Dudik M (2008). Modeling of species distributions with Maxent: new extensions and a comprehensive evaluation. Ecography.

[69] Piñeiro-Corbeira C, Barreiro R, Olmedo M, De la Cruz-Modino R (2020). Recreational snorkeling activities to enhance seascape enjoyment and environmental education in the Islas Atlánticas de Galicia National Park Spain. J Environ Manag.

[70] Planas M, Chamorro A, Quintas P, Vilar A (2008). Establishment and maintenance of threatened long-snouted seahorse, *Hippocampus guttulatus*, broodstock in captivity. Aquaculture.

[71] Planas M, Chamorro A, Paltrinieri A, Campos S, Nedelec K, Hernández-Urcera J (2020). Effect of diet on breeders and inheritance in Syngnathids: application of isotopic experimentally derived data to field studies. Mar Ecol Prog Ser.

[72] Planas M, Paltrinieri A, Dias-Carneiro MD, Hernández-Urcera J (2020). Effects of tissue preservation on carbon and nitrogen stable isotope signatures in syngnathid fishes and prey. Animals.

[73] Post DM (2002). Using stable isotopes to estimate trophic position: models, methods, and assumptions. Ecology.

[74] Post DM, Craig A, Layman D, Albrey Arrington D, Takimoto G, Quatrocchi J, Montaña CG (2007). Getting to the fat of the matter: models, methods and assumptions for dealing with lipids in stable isotope analyses. Oecologia.

[75] Puertos del Estado. http://www.puertos.es/es-es/oceanografia/Paginas/portus.aspx. 2017. Accessed 30 Dec 2019.

[76] Riera F, Pou S, Grau AM. La Ictiofauna. In: Alcover JJ, Ballesteros E, Fornós JJ, editors. Història Natural de l’Arxipèlag de Cabrera, Editorial Moll-CSIC;1993. p. 623-44.

[77] Rodil IF, Lastra M, López J (2009). Spatial variability of benthic macrofauna in the Ría of Vigo (NW Spain): effect of sediment type and food availability. Mar Biol Res.

[78] Román M, Rendal S, Fernández E, Méndez G (2018). Seasonal variability of the carbon and nitrogen isotopic signature in a *Zostera noltei* meadow at the NW Iberian Peninsula. Wetlands.

[79] Román M, Fernández E, Méndez G (2019). Anthropogenic nutrient inputs in the NW Iberian Peninsula estuaries determined by nitrogen and carbon isotopic signatures of *Zostera noltei* seagrass meadows. Mar Environ Res.

[80] Rumolo P, Bonnano A, Barra M, Fanelli E (2016). Spatial variations in feeding habits and trophic levels of two small pelagic fish species in the central Mediterranean Sea. Mar Environ Res.

[81] Sanna D, Biagi F, Alaya HB, Maltagliati F (2013). Mitochondrial DNA variability of the pipefish *Syngnathus abaster*. J Fish Biol.

[82] Scilipoti D. Studio della comunità ittica residente all’interno dello Stagnone di Marsala (Sicilia occidentale): Distribuzione delle specie e ripartizione delle risorse in dipendenza di habitat a diversa complessità strutturale. PhD Thesis Dissertation, University of Messina; 1998.

[83] Shokri MR, Gladstone W, Jelbart J (2009). The effectiveness of seahorses and pipefish (Pisces: Syngnathidae) as a flagship group to evaluate the conservation value of estuarine seagrass beds. Aquatic Conserv Mar freshw Ecosyst.

[84] Skóra KE. The broad-nosed pipefish. In: Głowaciński Z, editor. Polish Red Data Book of Animals. Vertebrates. Warszawa: PWRiL; 2001. p. 316–18.

[85] Smith TM, Hindell JS, Jenkins GP, Connolly RM (2008). Edge effects on fish associated with seagrass and sand patches. Mar Ecol Prog Ser.

[86] Sogabe A, Takagi M (2013). Population genetic structure of the messmate pipefish *Corythoichthys haematopterus* in the northwest pacific: evidence for a cryptic species. SpringerPlus.

[87] Spjotvoll E, Stoline MR (1973). An Extension of the T-Method of multiple comparison to include the cases with unequal sample sizes. J Am Stat Assoc.

[88] Tarnowska K, Sapota MR (2007). Presence of the broad-nosed pipefish (*Syngnathus typhle*) in coastal waters of the Gulf of Gdańsk. Int J Oceanogr Hydrobiol.

[89] Thayer GW, Murphey PL, LaCroix MW (1994). Responses of plant communities in Western Florida Bay to the die-off of seagrasses. Bull Mar Sci.

[90] Thayer GW, Powell AB, Hoss DE (1999). Composition of larval, juvenile, and small adult fishes relative to changes in environmental conditions in Florida bay. Estuaries.

[91] UNESCO. World Heritage candidate. https://whc.unesco.org/en/tentativelists/6286/. 2019. Accessed 13 Dec 2019.

[92] Valdés JL, Román MR, Álvarez-Ossorio MT, Gauzens AL, Miranda A (1990). Zooplankton composition and distribution off the coast of Galicia. Spain J Plankton Res.

[93] Valladares S, Planas M (2012). Non-lethal dorsal fin sampling for stable isotope analysis in seahorses. Aquatic Ecol.

[94] Valladares S, Soto DX, Planas M (2016). Dietary composition of endangered seahorses determined by stable isotope analysis. Mar Freshw Res.

[95] Villegas-Ríos D, Alós J, March D, Palmer M, Mucientes G, Saborido-Rey F (2013). Home range and diel behavior of the ballan wrasse, *Labrus bergylta*, determined by acoustic telemetry. J Sea Res.

[96] Vincent ACJ, Berglund A, Ahnesjö I (1995). Reproductive ecology of five pipefish species in one eelgrass meadow. Environ Biol Fish.

[97] Vincent AC, Foster SJ, Koldewey HJ (2011). Conservation and management of seahorses and other Syngnathidae. J Fish Biol.

[98] Vives F. Aspectes hidrgràfiques i plantónics dekl voltants de l’Arxipèlag de Cabrera. In: Alcover JJ, Ballesteros E, Fornós JJ, editors. Història Natural de l’Arxipèlag de Cabrera. Editorial Moll-CSIC; 1993. p. 487–502.

[99] Vizzini S, Mazzola A (2004). The trophic structure of the pipefish community (Pisces Syngnathidae) from a Western Mediterranean seagrass meadow based on stable isotope analysis. Estuaries.

[100] Wilson AB, Vincent A, Ahnesjo I, Meyer A (2001). Male pregnancy in seahorses and pipefishes (Family Syngnathidae): rapid diversification of paternal brood pouch morphology inferred from a molecular phylogeny. J Hered.

[101] Woodall LC, Koldewey HJ, Boehm JT, Shaw W (2015). Past and present drivers of population structure in a small coastal fish, the European long snouted seahorse *Hippocampus guttulatus*. Conserv Genet.

[102] Woodall LC, Otero-Ferrer F, Correia M, Curtis J (2018). A synthesis of European seahorse taxonomy, population structure, and habitat use as a basis for assessment, monitoring and conservation. Mar Biol.

[103] WoRMS Editorial Board. World Register of Marine Species. http://www.marinespecies.org (2020). Accessed 22 Dec 2019.

[104] Wunder MB, West JB, Bowen GJ, Dawson TE, Tu KP (2010). Using isoscapes to model probability surfaces for determining geographic origins. Isoscapes: understanding movement, pattern, and process on earth through isotope mapping.

[105] Yildiz T, Uzer U, Karakulak FS (2015). Preliminary report of a biometric analysis of greater pipefish *Syngnathus acus* Linnaeus, 1758 for the western Black Sea. Turk J Zool.

