# Supplementary material for: A multidisciplinary approach to identify priority areas for the monitoring of a vulnerable family of fishes in Spanish Marine National Parks
Source: BMC Ecol Evol. 2021 Jan 21;21:4. doi: 10.1186/s12862-020-01743-z (PMC7853308; doi:10.1186/s12862-020-01743-z)
Supplement: Supplementary file 2 — Additional file 2: Textural and compositional parameters of sediment (PNIA), general characteristics of macroalgal species and communities (PNIA), haplotypes detected in syngnathids (PNIA and PNAC), PCO Principal coordinates ordination for seaweed assemblages and syngnathids (PNIA), and length–weight relationships in syngnathids (PNIA). [file 12862_2020_1743_MOESM2_ESM.pdf]

A multidisciplinary approach to identify priority areas for the conservation of a vulnerable family of fishes in Spanish Marine National Parks

Miquel Planas, Cristina Piñeiro-Corbeira, Carmen Bouza, Inés Castejón-Silvo, Manuel Vera, Marcos Regueira, Verónica Ochoa, Ignacio Bárbara, Jorge Terrados, Alexandro Chamorro, Rodolfo Barreiro, Jorge Hernández-Urcera, Irene Alejo, Miguel Nombela, Manuel Enrique García, Belén G. Pardo, Viviana Peña, Pilar Díaz, Javier Cremades, Beatriz Morales-Nin

Department of Ecology and Marine Resources, Instituto de Investigaciones Marinas (IIM-CSIC), Eduardo Cabello 6, 36208 Vigo (Spain). e-mail: mplanas@iim.csic.es

Supplementary material

Table S1. PNIA - Mean values of the textural and compositional parameters of sediment along transects TR1 – TR10 (Spring and summer 2016 surveys in Cíes Archipelago).). MS - Moderately sorted; PS - Poorly sorted. n - Number of samples analyzed.

Season	Transect	n	$CaCO_3$ %	Gravel %	Sand %	Mud %	Mean (µm)		Sorting		Skewness
Spring	1	2	45.0	13.4	86.5	0.2	803	coarse sand	0.77	MS	0.05
	2	5	58.5	13.1	86.5	0.3	791	coarse sand	1.10	PS	-0.21
	3	15	79.0	11.5	84.8	3.7	790	coarse sand	1.11	PS	-0.11
	4	8	62.1	13.5	86.4	0.1	797	coarse sand	0.95	MS	-0.11
	5	7	56.2	12.0	65.7	22.3	276	medium sand	2.32	PS	-0.01
	6	3	48.0	3.8	96.2	0.0	283	medium sand	0.83	MS	-0.28
	7	6	28.8	7.3	92.7	0.1	374	medium sand	0.95	MS	-0.36
	8	4	70.7	7.9	92.3	0.0	860	coarse sand	0.64	MS	-0.10
	9	2	80.1	8.6	91.0	0.5	683	coarse sand	1.06	PS	0.06
Summer	3	4	80.7	1.3	98.6	0.1	382	medium sand	0.69	MS	-0.11
	4	9	38.4	11.2	88.7	0.2	701	coarse sand	0.89	MS	-0.22
	6	4	48.6	7.2	91.2	1.6	395	medium sand	1.1	PS	-0.21
	8	3	71.7	1.1	98.5	0.3	372	medium sand	0.87	MS	-0.05
	10	4	47.8	5.3	94.6	0.1	462	medium sand	0.83	MS	-0.29
		**Mean**	**58.3**	**8.4**	**89.5**	**2.1**	**569**	**coarse sand**	**1.01**	**PS**	**-0.14**

Table S2. PNIA - Mean values of the textural and compositional parameters of sediment along transects with presence of syngnathids (Spring and summer 2016 surveys in Cíes Archipelago). MS - Moderately sorted; PS - Poorly sorted. n - Number of samples analyzed.

Season	Transect	n	$CaCO_3$ %	Gravel %	Sand %	Mud %	Mean (µm)		Sorting		Skewness
Spring	2	1	47.0	16.6	83.0	0.1	679	coarse sand	1.41	MS	-0.41
	3	6	79.0	10.2	89.8	0.0	835	coarse sand	0.91	MS	-0.16
	5	1	19.8	1.8	98.2	-	413	medium sand	0.57	MS	0.01
	6	1	54.0	5.9	94.1	-	285	medium sand	0.94	MS	-0.39
Summer	3	1	80.4	0.1	99.8	-	204	medium sand	0.45	MS	-0.03
	4	2	39.1	10.4	89.6	-	808	coarse sand	0.63	MS	-0.20
	10	2	39.2	6.8	93.2	0.1	516	coarse sand	0.77	MS	-0.41
		**Mean**	**51.2**	**7.4**	**92.5**	**0.1**	**534**	**coarse sand**	**0.8**	**MS**	**-0.2**

Table S3. PNIA - Macroalgal species with medium-high abundances in transects TR1 to TR10 (spring and summer 2016). B, R and G – Brown, red and green algae, respectively. FO – Frequency of occurrence (%).

	Transect	TR1	TR2	TR3	TR4	TR5	TR6	TR7	TR8	TR9	TR10	
	**Species (Total=55)**	**n=7**	**n=20**	**n=31**	**n=29**	**n=30**	**n=35**	**n=12**	**n=38**	**n=31**	**n=22**	**FO**
B	*Cutleria multifida* phase *Aglaozonia*			+		+	+					30
B	*Carpodesmia tamariscifolia*			+		+					+	30
B	*Carpomitra costrata*						+		+	+		30
B	*Cladostephus spongiosus*		+	+		+			+	+	+	60
B	*Colpomenia peregrina*			+	+	+	+				+	50
B	*Cutleria adspersa*						+			+		20
B	*Cutleria multifida*			+		+						20
B	*Dictyopteris lucida*						+		+	+		30
B	*Dictyopteris polypodoides*			+	+	+	+		+			50
B	*Dictyota dichotoma*	+	+	+	+	+	+	+	+	+	+	100
B	*Halidrys siliquosa*							+	+	+	+	40
B	*Halopteris filicina*				+				+	+		30
B	*Halopteris scoparia*		+	+								20
B	*Laminaria ochroleuca*			+	+		+		+	+		50
B	*Padina pavonica*		+	+	+	+	+	+	+		+	80
B	*Phyllariopsis brevipes* subsp *pseudopurpurascens*								+			10
B	*Phyllariopsis purpurascens*						+		+	+		30
B	*Saccorhiza polyschides*	+	+	+	+	+	+		+	+	+	90
B	*Sargassum muticum*			+	+	+	+				+	50
B	*Taonia atomaria*			+	+	+	+	+		+		60
B	*Treptacantha baccata*		+	+	+	+	+	+	+		+	80
B	*Treptacantha nodicaulis*				+	+					+	30
B	*Treptacantha usneoides*		+	+	+	+	+		+		+	70
B	*Undaria pinnatifida*			+	+	+	+		+		+	60
B	*Zanardinia typus*								+			10
R	*Asparagopsis armata* phase *Falkenbergia rufolanosa*	+	+	+	+	+	+	+	+	+		90
R	*Acrosorium ciliolatum*					+					+	20
R	*Ahnfeltia plicata*									+		10
R	*Asparagopsis armata*	+	+	+	+	+	+	+	+	+	+	100
R	*Bonnemaisonia asparagoides*	+								+		20
R	*Chondracanthus (acicularis/teedei)*			+	+	+	+			+		50
R	*Chondria (coerulescens/scintillans)*			+	+	+	+		+	+		60
R	*Corallina/Ellisolandia/Jania spp.*		+	+	+	+	+	+	+	+		80
R	*Cruoria pellita*								+	+		20
R	*Dasysiphonia japonica*			+		+						20
R	*Dilsea carnosa*								+	+		20
R	*Gelidium corneum*						+		+			20
R	*Gelidium spinosum*		+			+					+	30
R	*Gracilaria (gracilis/multipartita)*		+	+	+	+	+		+		+	70
R	*Kallymenia (reniformis/crouaniorum)*				+		+	+	+	+		50
R	*Lithophyllum (incrustans/hibernicum)*		+	+	+	+	+	+	+	+		80
R	*Mesophyllum expansum*	+	+	+	+			+	+	+		70

		TR1	TR2	TR3	TR4	TR5	TR6	TR7	TR8	TR9	TR10	
	**Species (Total=55)** **Transect**	**n=7**	**n=20**	**n=31**	**n=29**	**n=30**	**n=35**	**n=12**	**n=38**	**n=31**	**n=22**	**FO**
R	*Peyssonnelia (coriacea/dubyi/immersa)*			+	+	+	+		+	+	+	70
R	*Phymatolithon (calcareum/lusitanicum)*		+		+							20
R	*Plocamium cartilagineum*		+		+	+	+		+	+	+	70
R	*Plocamium raphelisianum*								+	+		20
R	*Pterosiphonia complanata*						+		+			20
R	*Scinaia interrupta*		+				+		+			30
R	*Sphaerococcus coronopifolius*								+			10
R	*Stenogramme interrupta*		+	+	+	+	+					50
G	*Codium adhaerens*								+	+		20
G	*Codium fragile*			+		+	+		+	+	+	60
G	*Codium tomentosum*		+	+	+		+		+	+	+	70
G	*Codium vermilara*	+		+	+		+	+	+	+	+	80
G	*Ulva (rigida/australis)*		+	+	+	+	+		+	+	+	80

Table S4. PNIA - Variable sites for the haplotypes detected in *Syngnathus acus* and *Hippocampus guttulatus* respect to reference sequences AF356040 and AF192664, respectively. Dots denote identical sites.

	11
	2555789900
	148566766834
	057589023731
S_acus_AF356040	CTTAGCCCAACC
Cytb_SA01	....A....G..
Cytb_SA02	.....T.T...T
Cytb_SA07	.......T..TT
Cytb_SA10	...G.T.TT..T
Cytb_SA11	.....TTT...T
Cytb_SA13	....
Cytb_SA14	....A.......
Cytb_SA16	..C.........
Cytb_SA17	TC...T.T...T

	33445
	77684
	36956
AF192664	GGATG
Cytb_HG01	AAG.T
Cytb_HG02	AA.GT
Cytb_HG03	AA..T

Table S5. PNAC - Variable sites for the haplotypes detected in *Syngnathus abaster* respect to the reference sequence AF356040. Dots denote identical sites.

		1111111122	2222222233	3333333333	3344444444	4444444555	5555555555	5556666666	6777777777	7777777888	8888888999	9999999999	0000000000	000011111
	12236899	0012227802	4447778902	2444555677	7801123456	6677889001	3444556678	8890123357	9001122334	4566889001	2245589000	0134566899	0233356678	999900114
	4901809436	2543684078	0690365762	4258278925	8721465762	5847392173	4069581760	2376840948	3281206257	8958035140	2565621036	7865909789	2023895990	027814064
S_acus_AF356040	GCCCACACGT	GAGAACTTCG	TGCTCCTTAC	ATCGAACAAT	CAGAAGTTGC	CCCCCGCTAC	CCCCAACCCA	CACGTTGACC	AGCTACTTCC	TGCCCTTTCG	CGATACTTGA	CTAGTGCACG	TAGCAATGCA	CATCCACGG
Cytb_SAb01	ATTTGTCAAC	ATAGCTCCTA	CATCTTCCGT	GCTAGGTCGC	TGAGGACAAT	TTTTTAACCT	TTTTCCTATG	ACTACCAGTT	GAACGTCCTT	CATTTCCCTA	TACC.TCCAG	TCGACCTCTC	CCAGCGAATG	TGCTTGTAA
Cytb_SAb02	ATTTGTCAAC	ATAGCTCCTA	CATCTTCCGT	GCTAGGTCGC	TGAGGACAAT	TTTTTAACCT	TTTTCCTATG	ACTACCAGTT	GAACGTCCTT	CATTTCCCTA	TACCCTCCAG	TCGACCTCTC	CCAGCGAATG	TGCTTGTAA

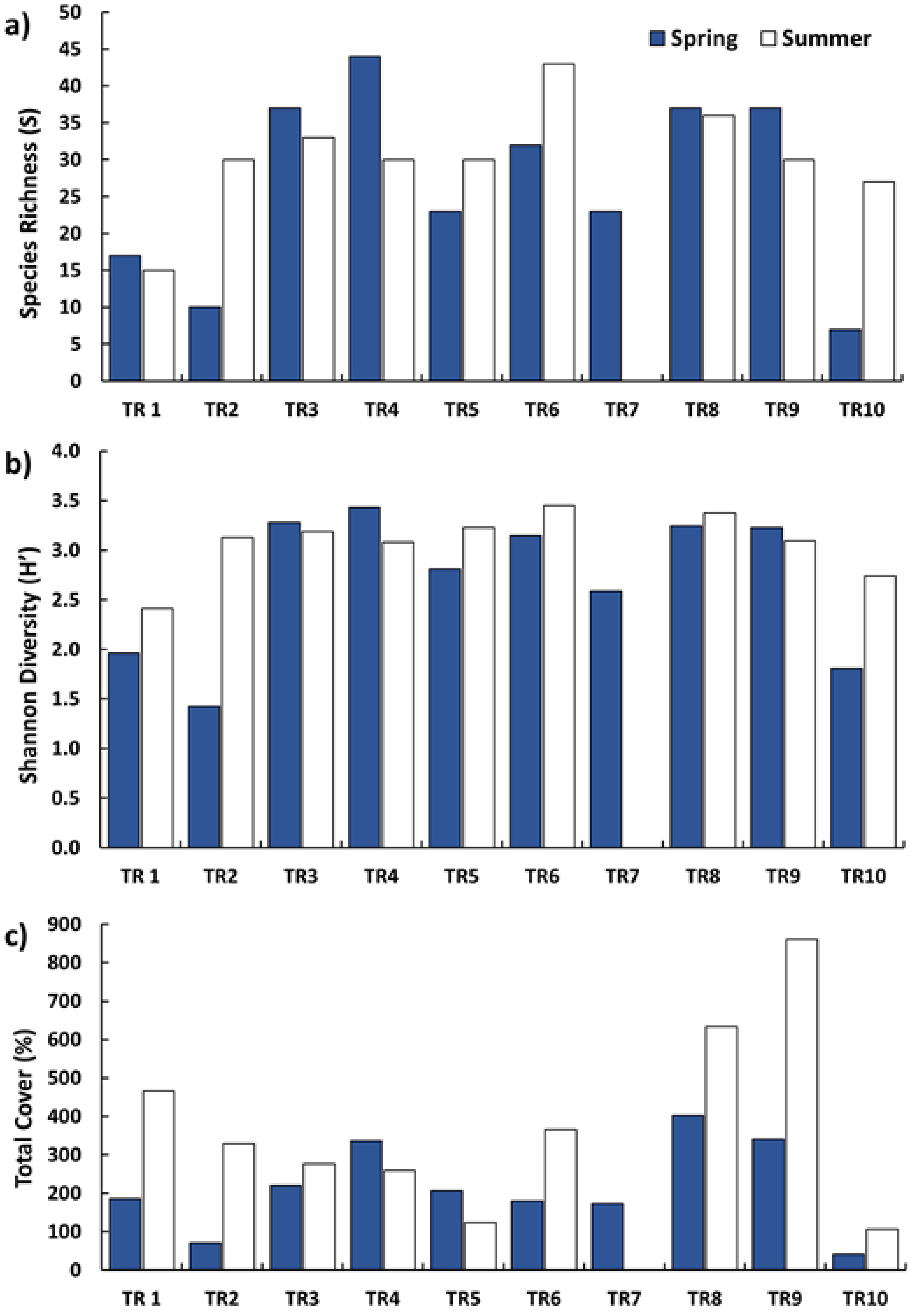

Figure S1. PNIA - Total cover (%), diversity (H') and species richness (S) of seaweeds in transects TR1 – TR10 surveyed in Cíes Archipelago in spring and summer 2016.

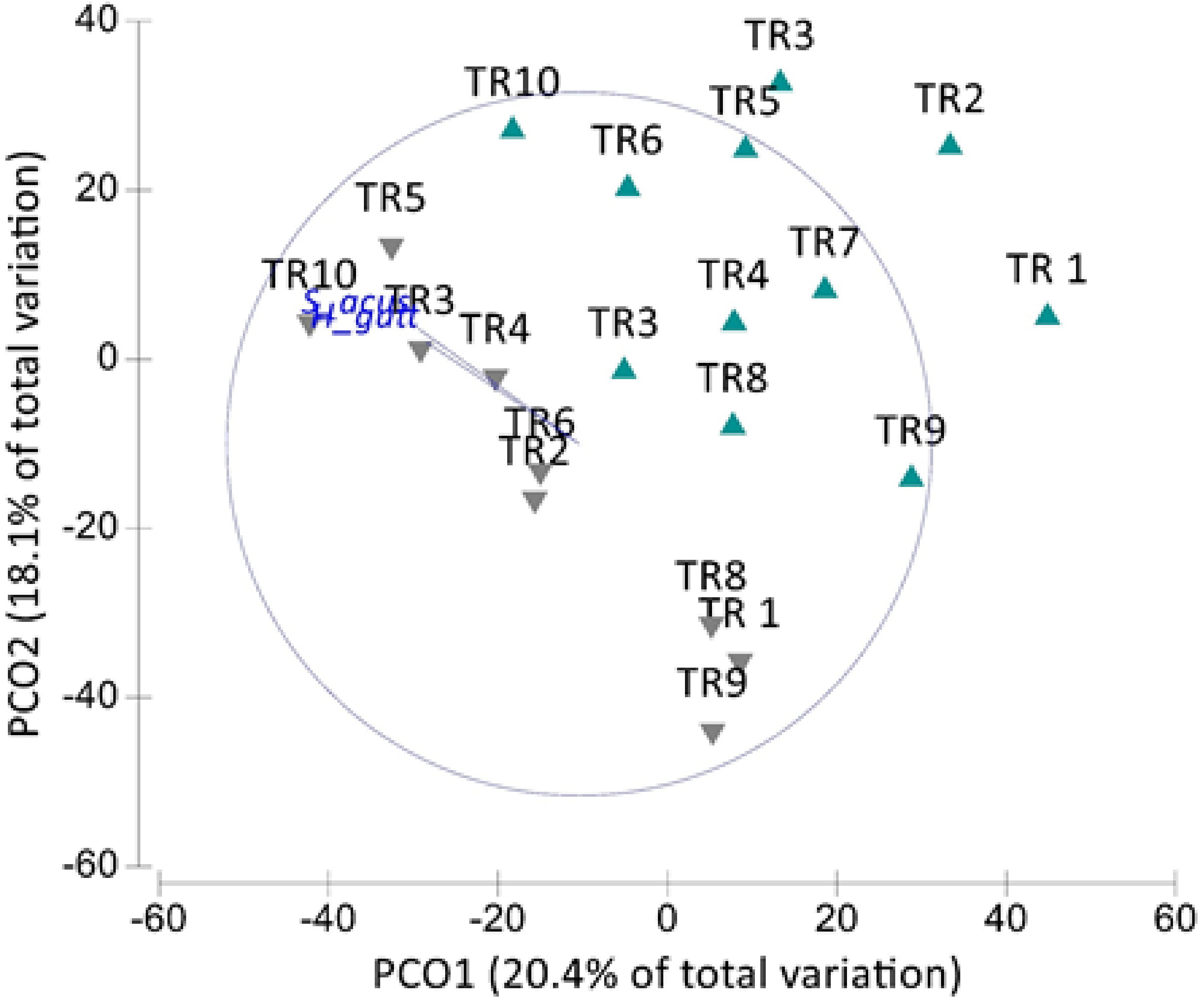

Figure S2. PNIA - Principal coordinates ordination of samples for Transect x Season pairwise combinations of seaweed assemblage in Cíes Archipelago on spring (green) and summer (grey). Overlay vectors are syngnathids whose abundance has a Spearman correlation >0.65 with any axis.

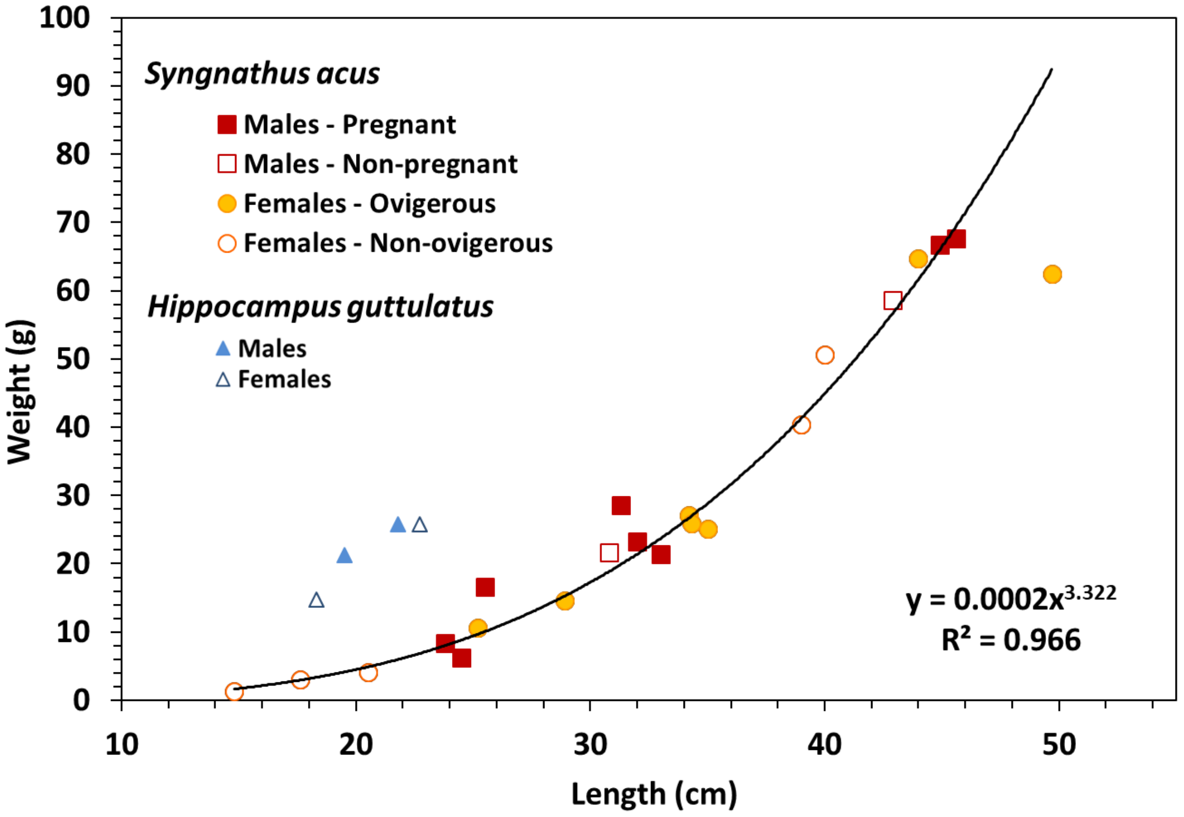

Figure S3. PNIA - Length-weight relationships in *S. acus* and *H. guttulatus* from 2016 surveys in Cíes Archipelago. Regression equation provided for *S. acus*.

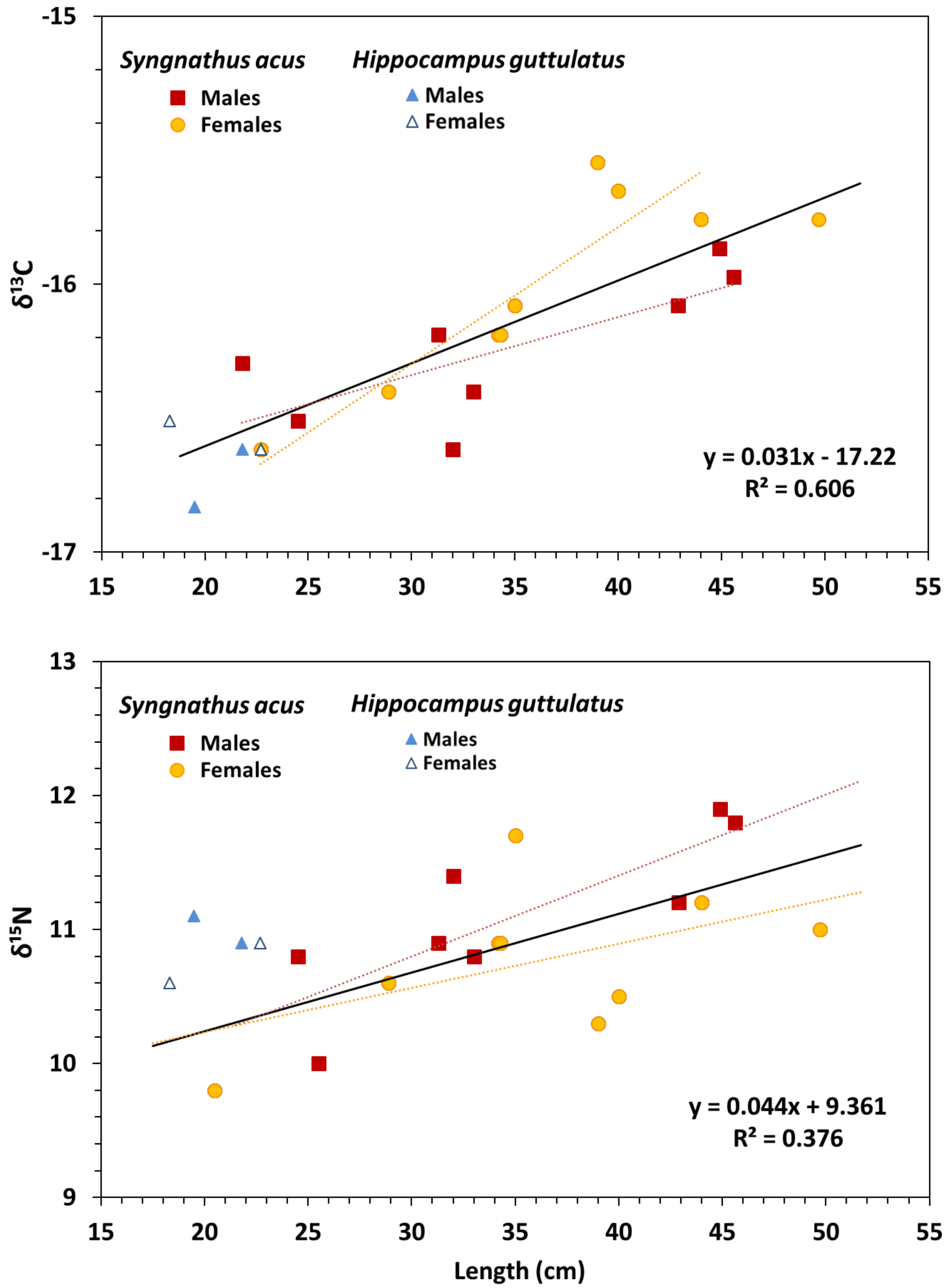

Figure S4. PNIA - Stable isotopes-length relationships in *S. acus* and *H. guttulatus* from 2016 surveys in Cíes Archipelago. Dotted lines: Regression lines for *S. acus* males (red) and females (yellow). Regression equation provided for all *S. acus* specimens (continuous line).
