# Supplementary material for: A multidisciplinary approach to identify priority areas for the monitoring of a vulnerable family of fishes in Spanish Marine National Parks
Source: BMC Ecol Evol. 2021 Jan 21;21:4. doi: 10.1186/s12862-020-01743-z (PMC7853308; doi:10.1186/s12862-020-01743-z)
Supplement: Supplementary file 3 — Additional file 3: Illustrations of main species (medium–high abundance) of macroalgae and bathymetric zonation of seaweed assemblages in transects surveyed on Cíes Archipelago (PNIA). [file 12862_2020_1743_MOESM3_ESM.pdf]

Spanish Marine National Parks: Priority areas for the conservation of a vulnerable family of fishes

BMC Ecology

Miquel Planas, Cristina Piñeiro-Corbeira, Carmen Bouza, Inés Castejón-Silvo, Manuel Vera, Marcos Regueira, Verónica Ochoa, Ignacio Bárbara, Alexandro Chamorro, Jorge Terrados, Rodolfo Barreiro, Jorge Hernández-Urcera, Irene Alejo, Miguel Nombela, Manuel Enrique García, Belén G. Pardo, Viviana Peña, Pilar Díaz, Javier Cremades, Beatriz Morales-Nin

Department of Ecology and Marine Resources, Instituto de Investigaciones Marinas (IIM-CSIC), Eduardo Cabello 6, 36208 Vigo (Spain). e-mail: mplanas@iim.csic.es

Supplementary material

Figure S1. PNIA – Bathymetric zonation of vegetal assemblages in transect TR1 (Cíes Archipelago)

Figure S2. PNIA – Bathymetric zonation of seaweed assemblages in transect TR2 (Cíes Archipelago)

Figure S3. PNIA – Bathymetric zonation of seaweed assemblages in transect TR3 (Cíes Archipelago)

Figure S4. PNIA – Bathymetric zonation of seaweed assemblages in transect TR4 (Cíes Archipelago)

Figure S5. PNIA – Bathymetric zonation of seaweed assemblages in transect TR5 (Cíes Archipelago)

Figure S6. PNIA – Bathymetric zonation of seaweed assemblages in transect TR6 (Cíes Archipelago)

Figure S7. PNIA – Bathymetric zonation of seaweed assemblages in transect TR7 (Cíes Archipelago)

Figure S8. PNIA – Bathymetric zonation of seaweed assemblages in transect TR8 (Cíes Archipelago)

Figure S9. PNIA – Bathymetric zonation of seaweed assemblages in transect TR9 (Cíes Archipelago)

Figure S10. PNIA – Bathymetric zonation of seaweed assemblages in transect TR10 (Cíes Archipelago)

Figure S11. PNIA – Main species (medium-high abundances) of macroalgae in seaweed assemblages of Cíes Archipelago

Fig. S1

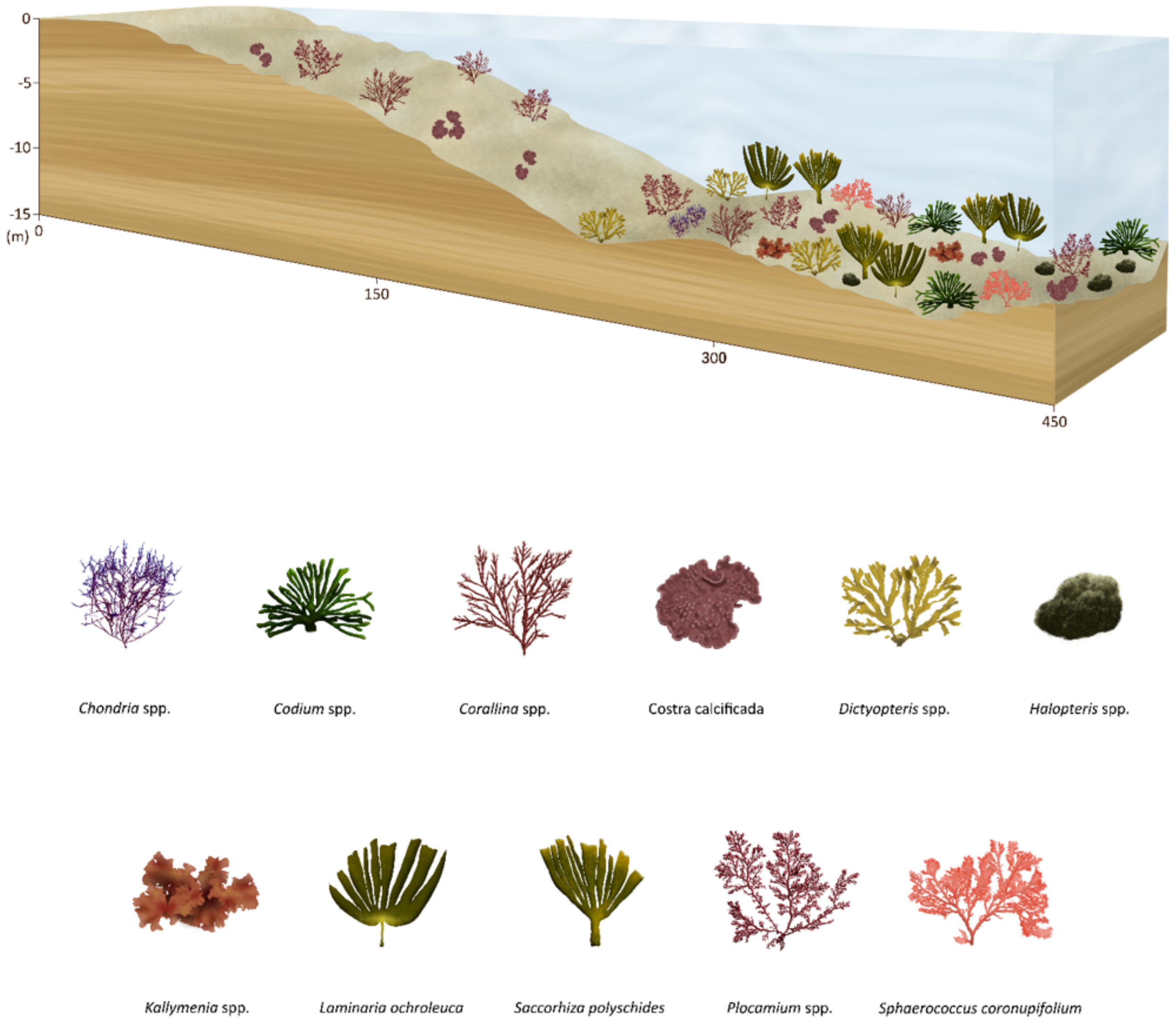
0
-5
-10
-15
(m)
0
150
300
450
Chondria spp.
Codium spp.
Corallina spp.
Costra calcificada
Dictyopteris spp.
Halopteris spp.
Kallymenia spp.
Laminaria ochroleuca
Saccorhiza polyschides
Plocamium spp.
Sphaerococcus coronupifolium

Fig. S2

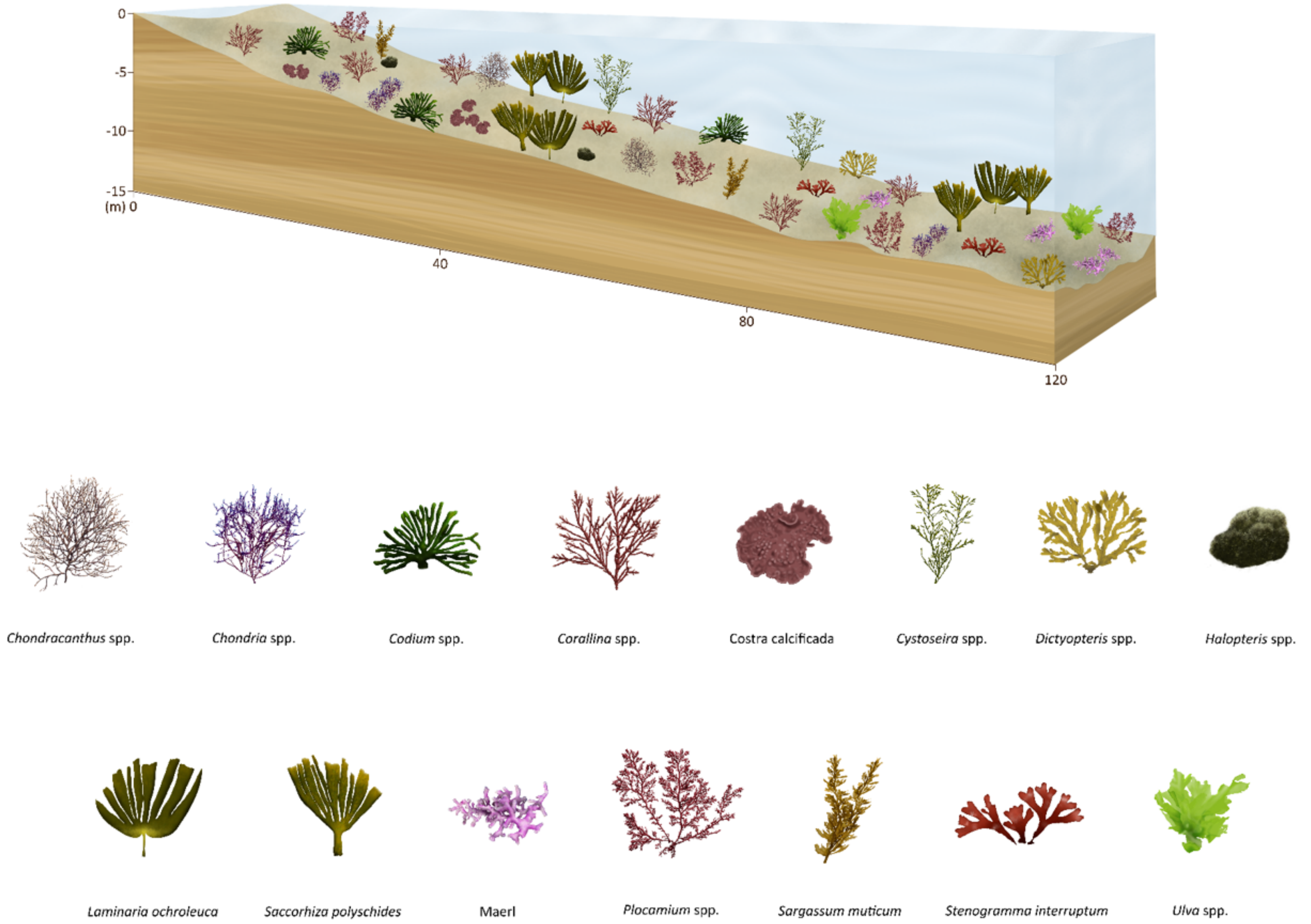
0
-5
-10
-15
(m) 0
40
80
120
Chondracanthus spp.
Chondria spp.
Codium spp.
Corallina spp.
Costra calcificada
Cystoseira spp.
Dictyopteris spp.
Halopteris spp.
Laminaria ochroleuca
Saccorhiza polyschides
Maerl
Plocamium spp.
Sargassum muticum
Stenogramma interruptum
Ulva spp.

Fig. S3

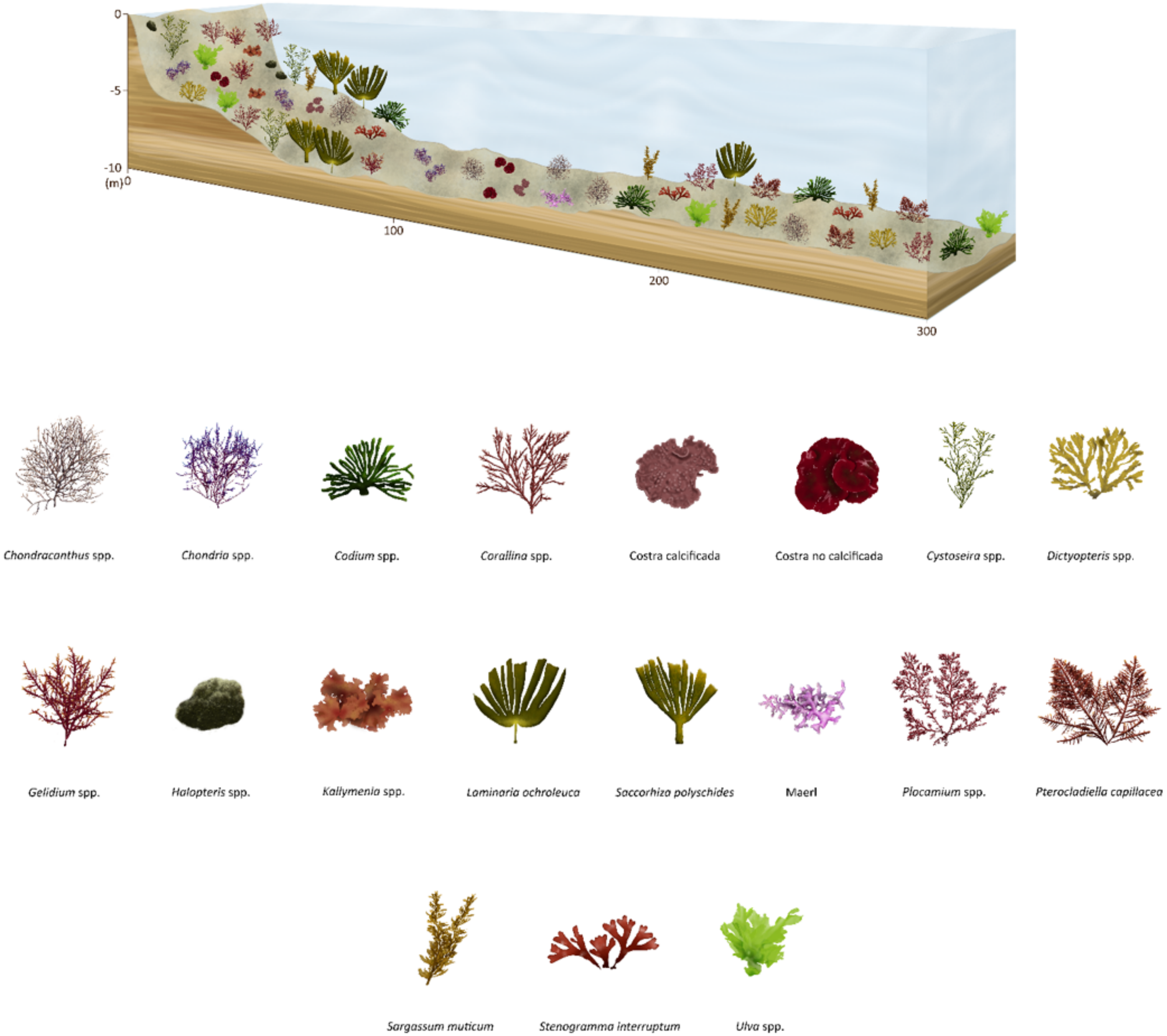

0
-5
-10
(m)
0
100
200
300
Chondracanthus spp.
Chondria spp.
Codium spp.
Corallina spp.
Costra calcificada
Costra no calcificada
Cystoseira spp.
Dictyopteris spp.
Gelidium spp.
Halopteris spp.
Kallymenia spp.
Laminaria ochroleuca
Saccorhiza polyschides
Maerl
Plocamium spp.
Pterocladiella capillacea
Sargassum muticum
Stenogramma interruptum
Ulva spp.

Fig. S4

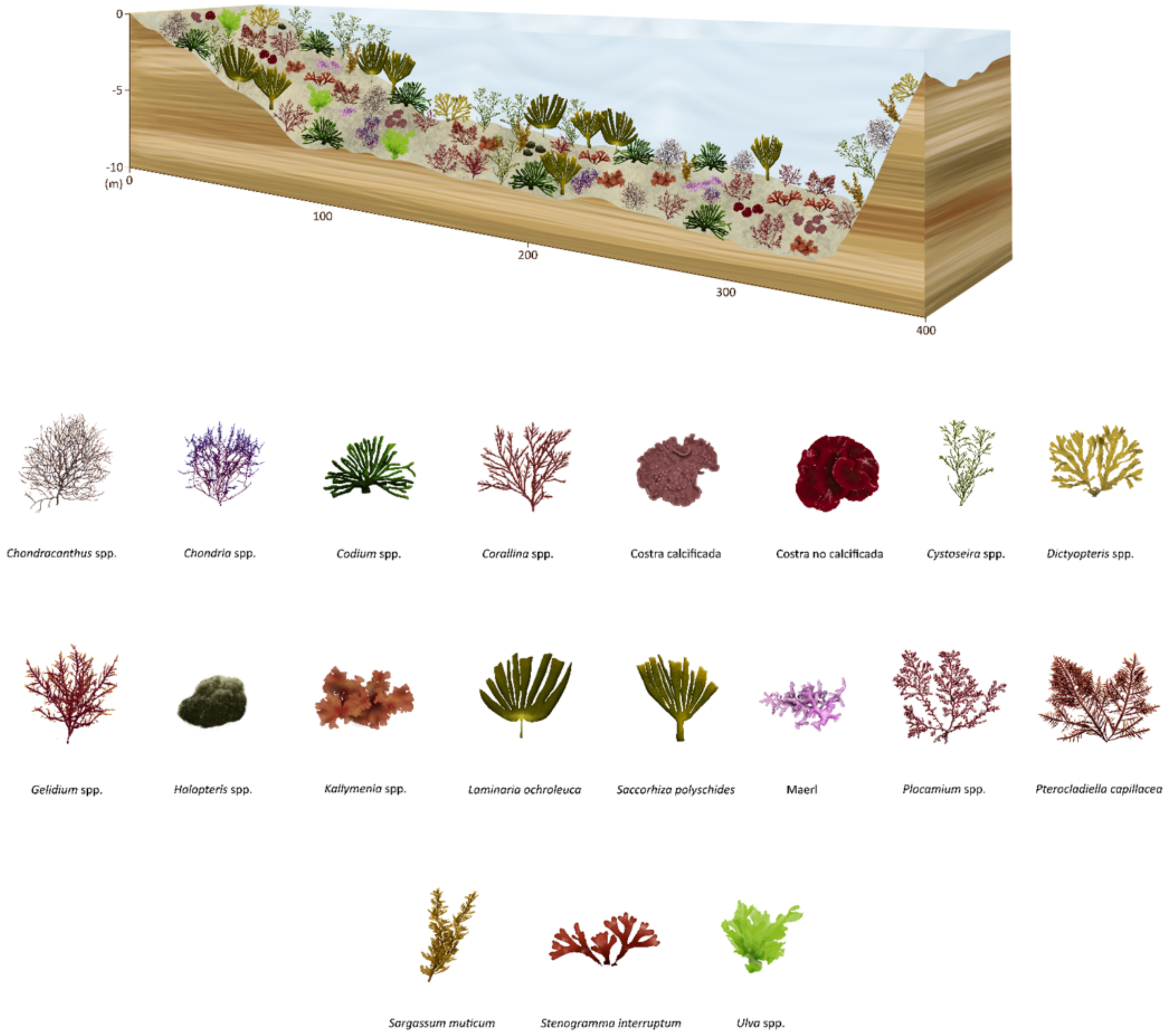

0
-5
-10
(m)
0
100
200
300
400
Chondracanthus spp.
Chondria spp.
Codium spp.
Corallina spp.
Costra calcificada
Costra no calcificada
Cystoseira spp.
Dictyopteris spp.
Gelidium spp.
Halopteris spp.
Kallymenia spp.
Laminaria ochroleuca
Saccorhiza polyschides
Maerl
Plocamium spp.
Pterocladiella capillacea
Sargassum muticum
Stenogramma interruptum
Ulva spp.

Fig. S5

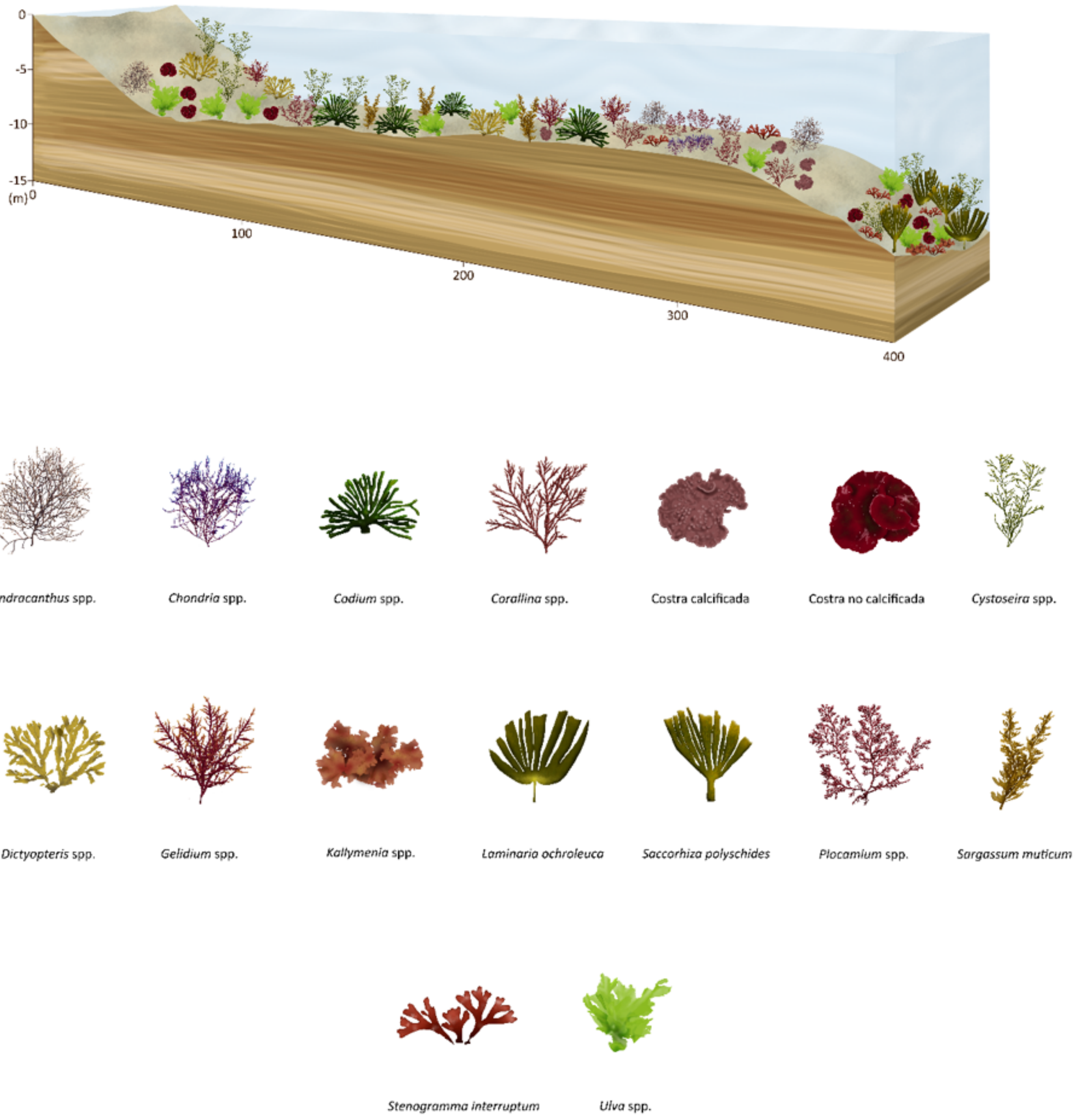
0
-5
-10
-15
(m)
0
100
200
300
400
Chondracanthus spp.
Chondria spp.
Codium spp.
Corallina spp.
Costra calcificada
Costra no calcificada
Cystoseira spp.
Dictyopteris spp.
Gelidium spp.
Kallymenia spp.
Laminaria ochroleuca
Saccorhiza polyschides
Plocamium spp.
Sargassum muticum
Stenogramma interruptum
Ulva spp.

Fig. S6

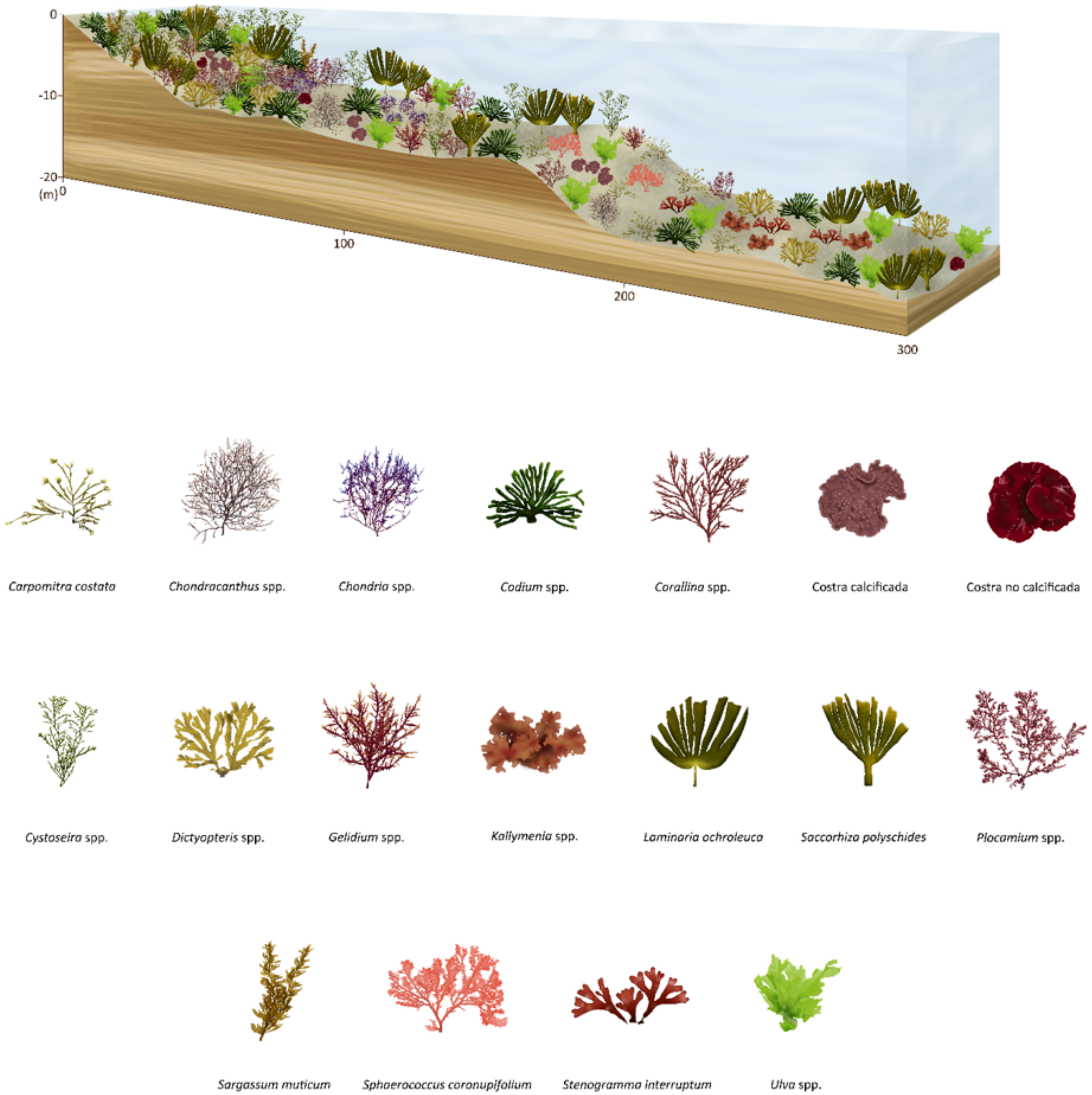
0
-10
-20
(m)
0
100
200
300
Carpomitra costata
Chondracanthus spp.
Chondria spp.
Codium spp.
Corallina spp.
Costra calcificada
Costra no calcificada
Cystoseira spp.
Dictyopteris spp.
Gelidium spp.
Kallymenia spp.
Laminaria ochroleuca
Saccorhiza polyschides
Plocamium spp.
Sargassum muticum
Sphaerococcus coronupifolium
Stenogramma interruptum
Ulva spp.

Fig. S7

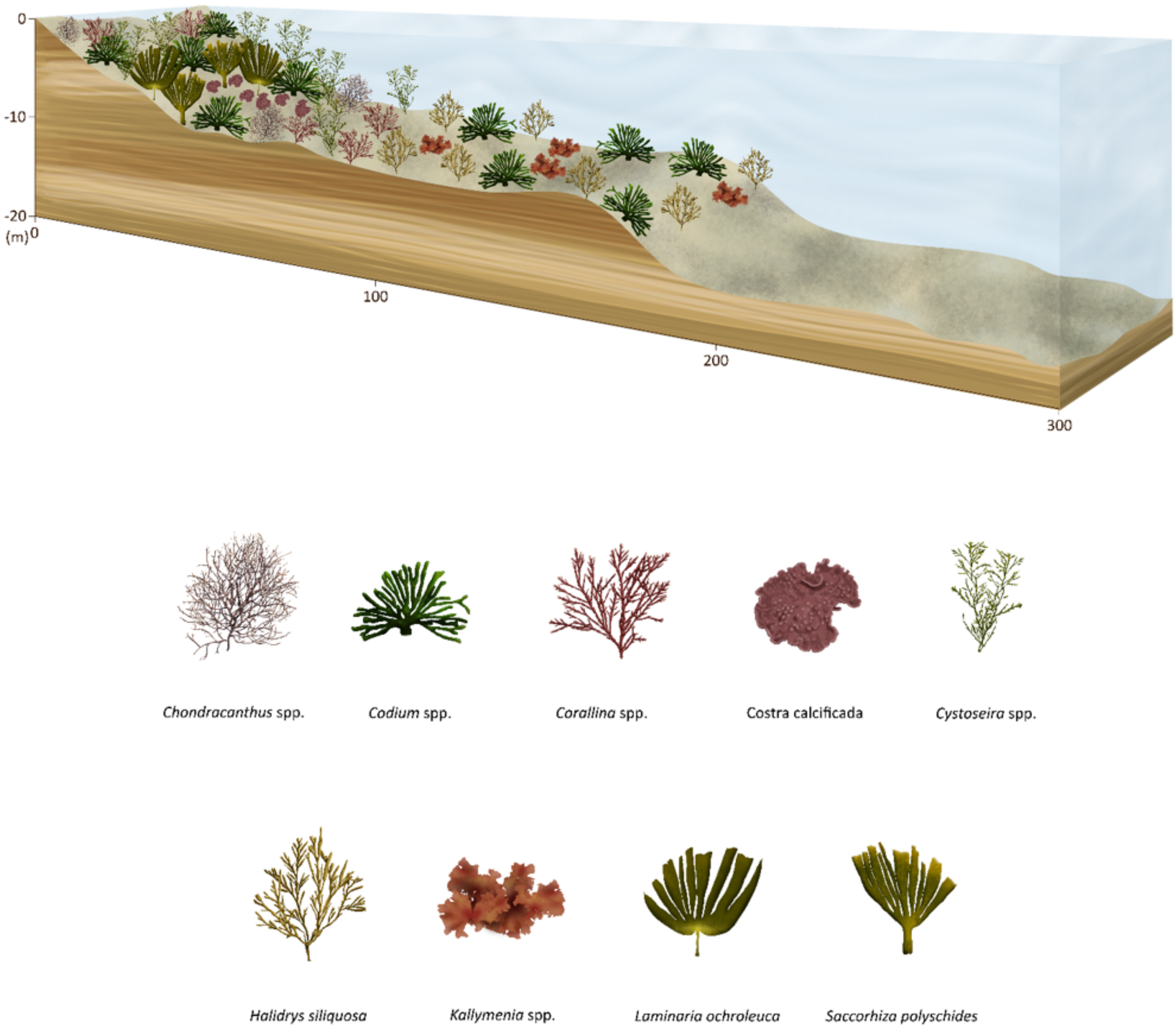

0
-10
-20
(m)
0
100
200
300
Chondracanthus spp.
Codium spp.
Corallina spp.
Costra calcificada
Cystoseira spp.
Halidrys siliquosa
Kallymenia spp.
Laminaria ochroleuca
Saccorhiza polyschides

Fig. S8

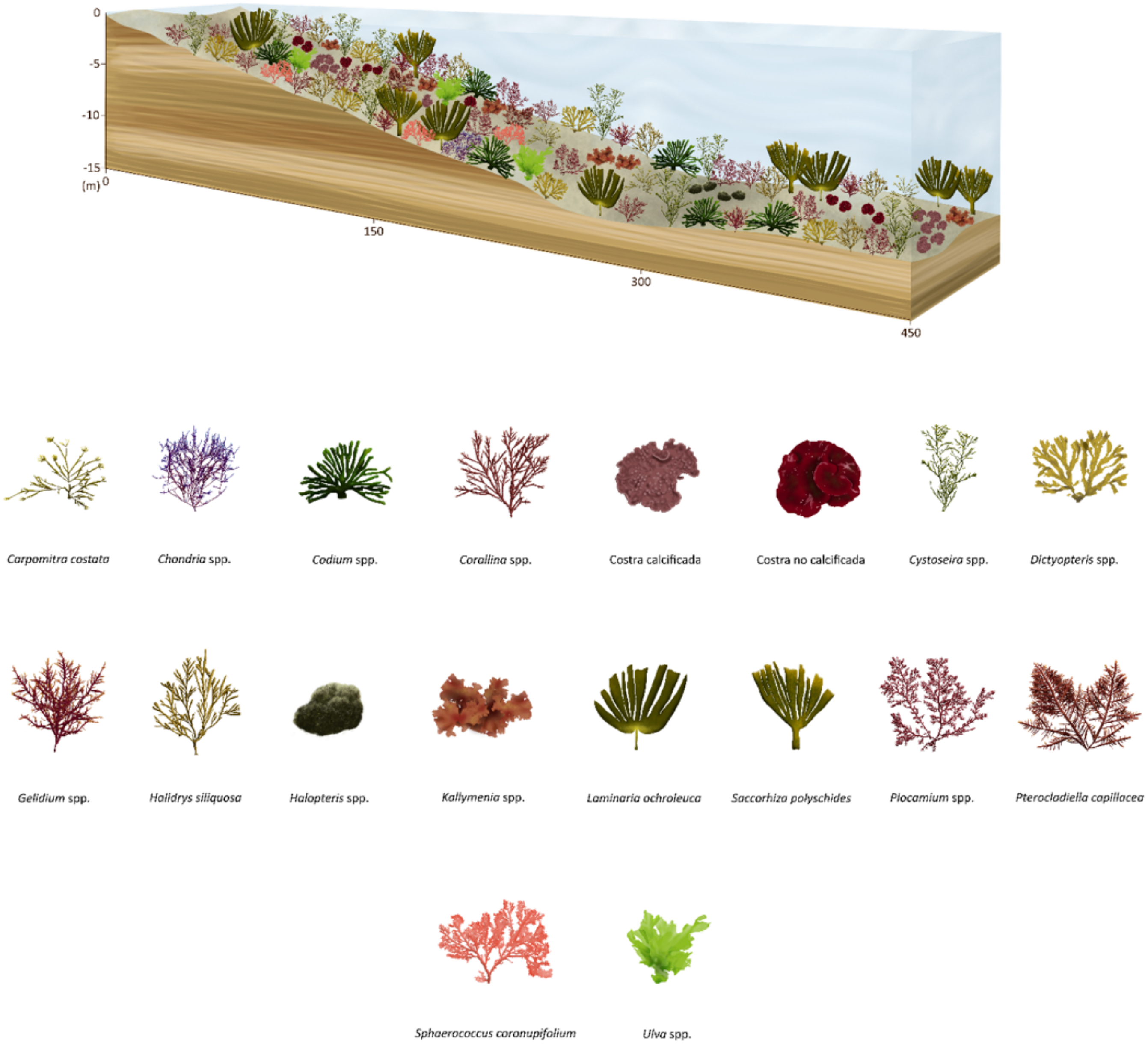
0
-5
-10
-15
(m)
0
150
300
450
Carpomitra costata
Chondria spp.
Codium spp.
Corallina spp.
Costra calcificada
Costra no calcificada
Cystoseira spp.
Dictyopteris spp.
Gelidium spp.
Halidrys siliquosa
Halopteris spp.
Kallymenia spp.
Laminaria ochroleuca
Saccorhiza polyschides
Plocamium spp.
Pterocladiella capillacea
Sphaerococcus coronupifolium
Ulva spp.

Fig. S9

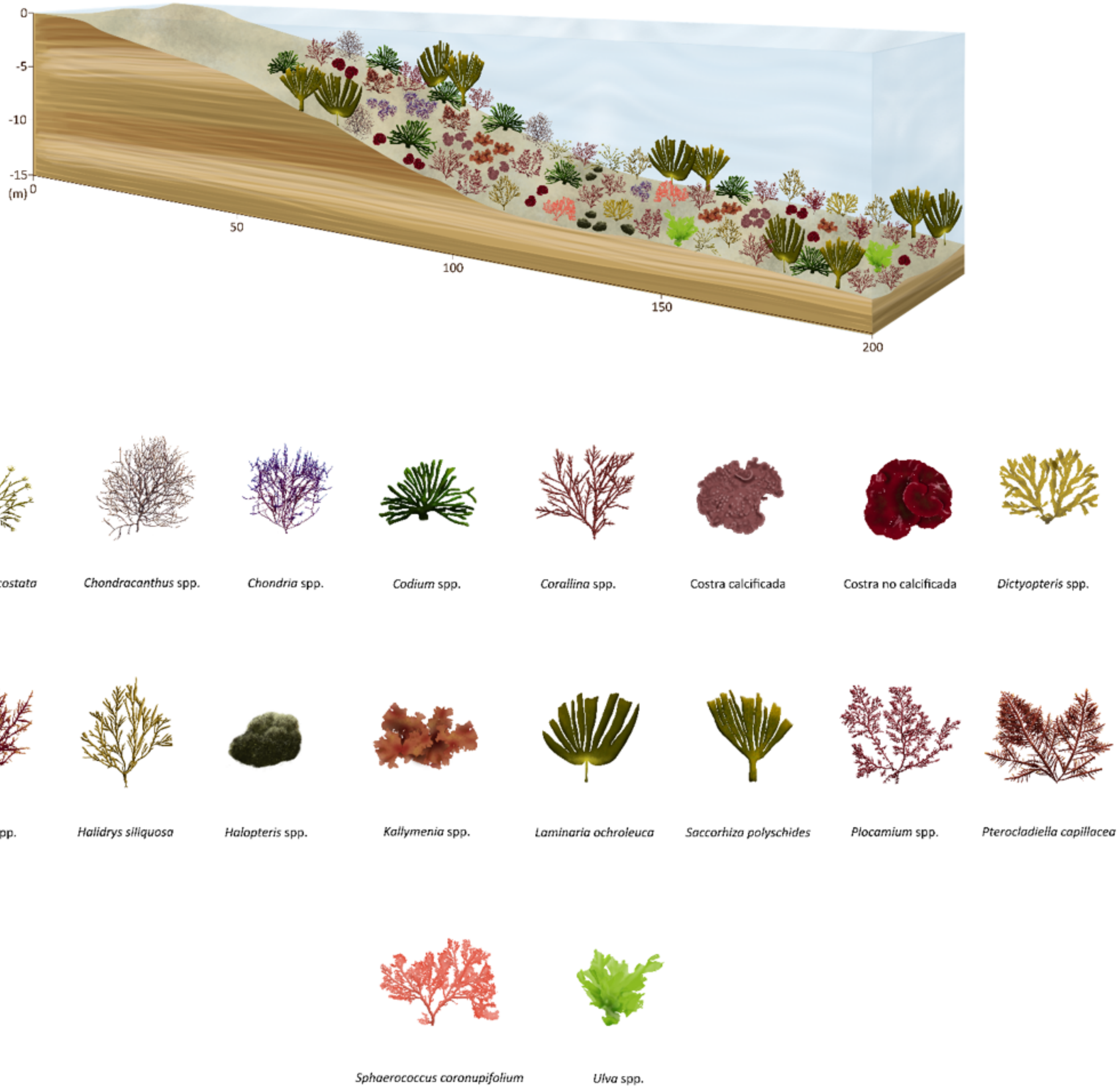
0
-5
-10
-15
(m)
0
50
100
150
200
Carpomitra costata
Chondracanthus spp.
Chondria spp.
Codium spp.
Corallina spp.
Costra calcificada
Costra no calcificada
Dictyopteris spp.
Gelidium spp.
Halidrys siliquosa
Halopteris spp.
Kallymenia spp.
Laminaria ochroleuca
Saccorhiza polyschides
Plocamium spp.
Pterocladiella capillacea
Sphaerococcus coronupifolium
Ulva spp.

Fig. S10

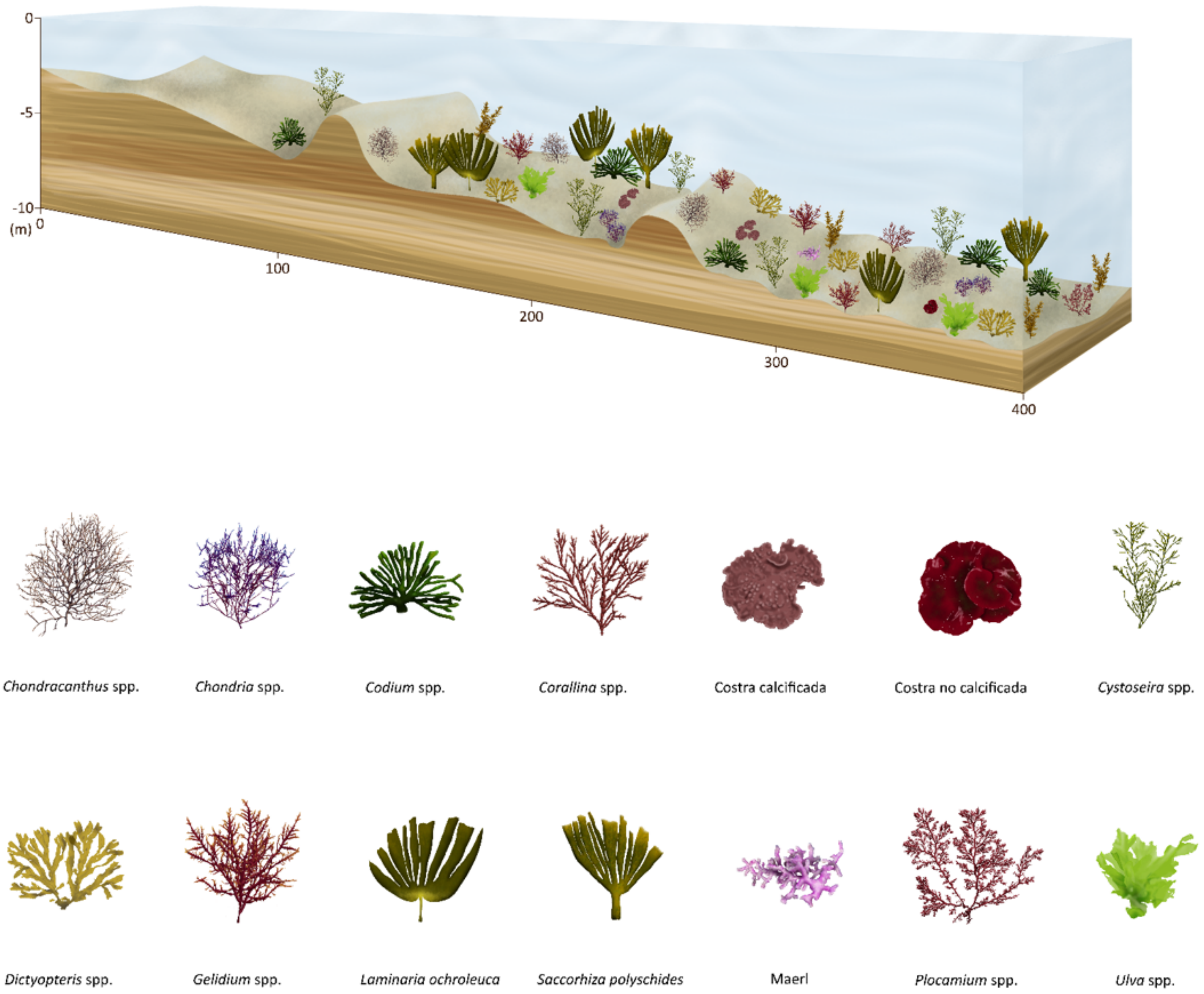

0
-5
-10
(m)
0
100
200
300
400
Chondracanthus spp.
Chondria spp.
Codium spp.
Corallina spp.
Costra calcificada
Costra no calcificada
Cystoseira spp.
Dictyopteris spp.
Gelidium spp.
Laminaria ochroleuca
Saccorhiza polyschides
Maerl
Plocamium spp.
Ulva spp.

Fig. S11

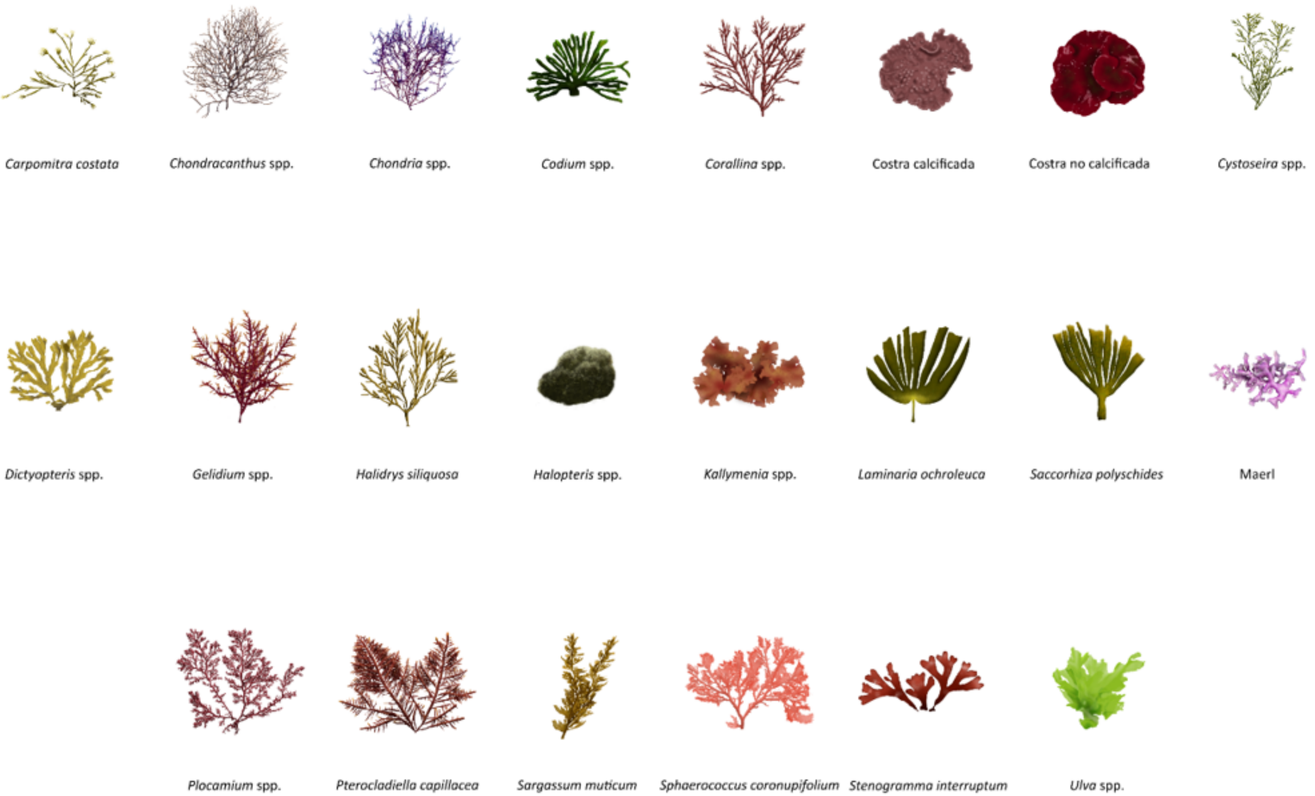

Carpomitra costata
Chondracanthus spp.
Chondria spp.
Codium spp.
Corallina spp.
Costra calcificada
Costra no calcificada
Cystoseira spp.
Dictyopteris spp.
Gelidium spp.
Halidrys siliquosa
Halopteris spp.
Kallymenia spp.
Laminaria ochroleuca
Saccorhiza polyschides
Maerl
Plocamium spp.
Pterocladiella capillacea
Sargassum muticum
Sphaerococcus coronupifolium
Stenogramma interruptum
Ulva spp.
